# Supplementary figures and images for: LuxS/AI-2 regulates phoP/phoQ by a non-canonical mechanism to enhance acid stress survival in Salmonella Typhimurium
Source: PLoS Pathog. 2026 May 28;22(5):e1014244. doi: 10.1371/journal.ppat.1014244 (PMC13218499; doi:10.1371/journal.ppat.1014244)

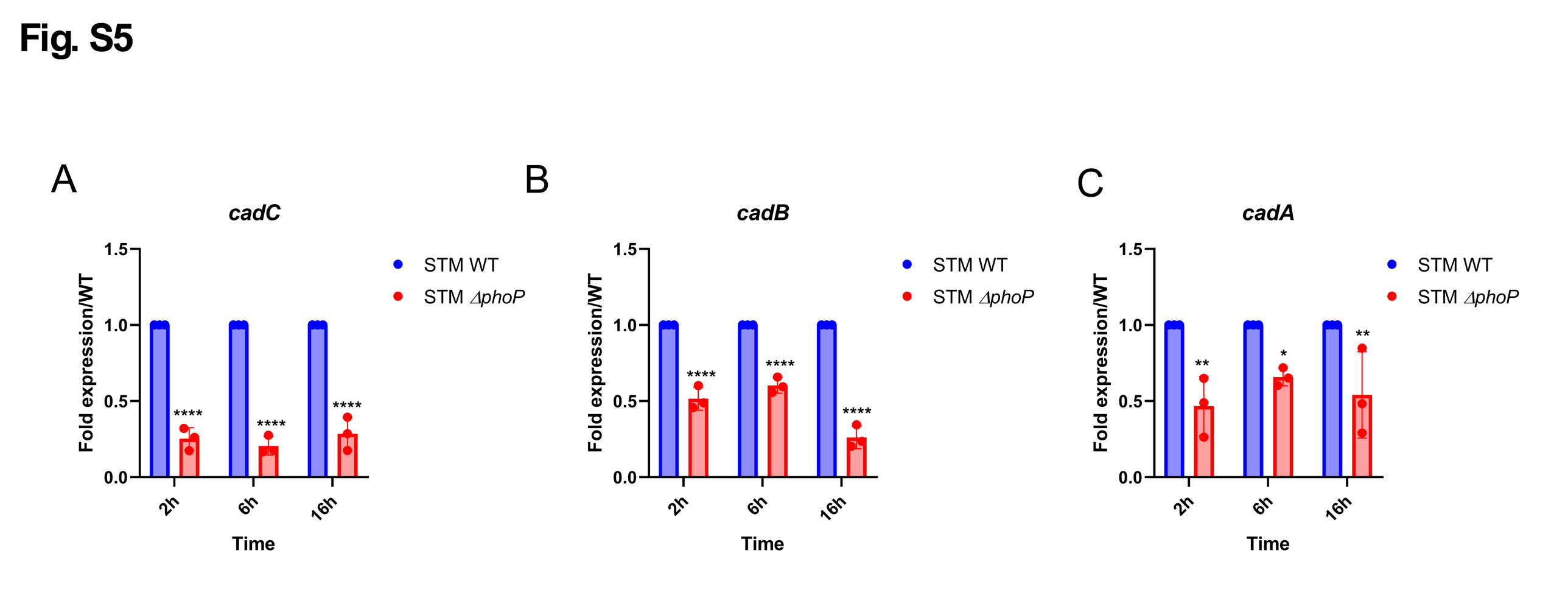

Supplement: S5 Fig — mRNA expression of (A) cadA, (B) cadB, and (C) cadC gene in STM WT and STM ∆phoP upon infection in RAW 264.7 macrophages. Two-way Anova was used to analyze the grouped data; p values **** p < 0.0001, *** p < 0.001, ** p < 0.01, * p < 0.05. All data are represented as mean ± SD from independent experiments (N = 3, n = 3). (TIF) [file ppat.1014244.s005.tif]

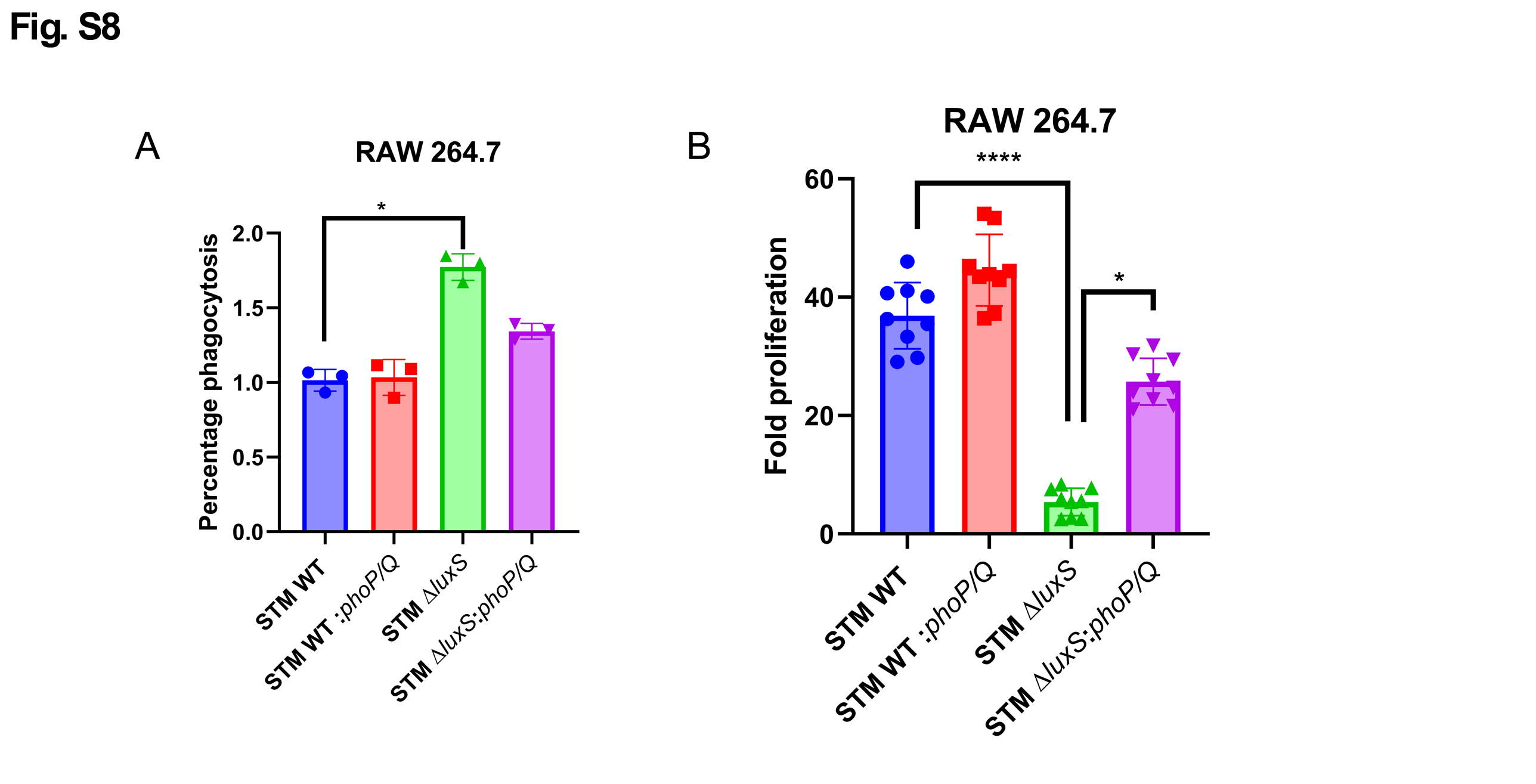

Supplement: S8 Fig — (A) Percentage phagocytosis, (B) Fold proliferation, of STM WT, STM WT: pQE60-phoP/phoQ, STM ∆luxS, STM ∆luxS: pQE60-phoP/phoQ upon infection in RAW 264.7. One-way ANOVA with Dunnett’s post-hoc test was used to analyze the data. (Data is from one experiment, representative of 3 independent experiments). (TIF) [file ppat.1014244.s008.tif]
