# Supplementary material for: LuxS/AI-2 regulates phoP/phoQ by a non-canonical mechanism to enhance acid stress survival in Salmonella Typhimurium
Source: PLoS Pathog. 2026 May 28;22(5):e1014244. doi: 10.1371/journal.ppat.1014244 (PMC13218499; doi:10.1371/journal.ppat.1014244)
Supplement: S1 Text — (DOCX) [file ppat.1014244.s012.docx]

**Supplementary information**

**LuxS/AI-2 regulates *phoP/phoQ* by a non-canonical mechanism to enhance acid stress survival in *Salmonella* Typhimurium**

Anmol Singh^1^, Abhilash Vijay Nair^1,#a,¶^, Shashanka Aroli^1¶^, Suman Das^2^, Subhrajit Karmakar^2^, R. S. Rajmani^3^, Santanu Mukherjee^3^, Umesh Varshney^1^, Dipshikha Chakravortty^1,4^*

^1^Department of Microbiology and Cell Biology, Division of Biological Sciences, Indian Institute of Science, Bengaluru, India

^2^Department of Organic Chemistry, Indian Institute of Science, Bengaluru, India

^3^Molecular Biophysics Unit, Indian Institute of Science, Bengaluru, India

^4^Adjunct Faculty, School of Biology, Indian Institute of Science Education and Research, Thiruvananthapuram, India

^#a^Present address: Department of Microbiology and Immunology, Vagelos College of Physicians and Surgeons, Columbia University, New York, NY 10032, USA

*Corresponding author: dipa@iisc.ac.in,

^¶^ These authors contributed equally.

Tel: +91 80 2293 2842,

Fax: +91 80 2360 2697

**Supplementary methods**

**Chemical synthesis of acetonide-protected (*S*)-4,5-dihydroxy-2,3-petanedione (DPD)**

Acetonide-protected (*S*)-4,5-dihydroxy-2,3-petanedione (DPD) was synthesized as described previously with modification (Sarkar and Makherjee, 2021)(1).

**A. General information:**

Infrared (FT-IR) spectra were recorded on a Bruker Alfa FT-IR, ν_max_ in cm^–1^ and the bands are characterized as broad (br), strong (s), medium (m), and weak (w). NMR spectra were recorded on Bruker Ultrashield spectrometer at 400 MHz (for ^1^H-NMR) and 100 MHz (for ^13^C-NMR). Chemical shifts are reported in ppm from tetramethylsilane with the solvent resonance as internal standard [CDCl_3_: δ 7.26, CD_3_OD: δ 3.31, (CD_3_)_2_SO: δ 2.50 for ^1^H-NMR and CDCl_3_: δ 77.16, CD_3_OD: δ 49.00, (CD_3_)_2_SO: δ 39.52 for ^13^C-NMR]. For ^1^H-NMR, data are reported as follows: chemical shift, multiplicity (s = singlet, d = doublet, dd = double doublet, ddd = doublet of doublet of doublets, t = triplet, q = quartet, sep = septet, br = broad, m = multiplet), coupling constants (Hz) and integration. High resolution mass spectrometry was performed on Waters XEVO G2-XS QTof instrument. Optical rotations were measured on a JASCO P-2000 polarimeter. Melting points were measured in open glass capillary using Büchi M-560 melting point apparatus. Enantiomeric ratios were determined by Shimadzu LC-20AD HPLC instrument and SPD-20A Diode Array Detector using stationary phase chiral columns (25 cm × 0.46 cm) in comparison with authentic racemic compounds.

**B. Procedure for the preparation of (*S*)-2,3-dihydroxypropanoate 2**

(*S*)-2,3-Dihydroxypropanoate **2** was prepared using modified literature procedure(2). To an ice-cooled solution of *L*-serine **1** (15 g, 142.7 mmol) in 30 ml H_2_O, H_2_SO_4_ (5M, 45 mL) was added, followed by slow addition of NaNO_2_ (15.3 g in 27.0 mL water). The reaction mixture was then stirred at ambient temperature for 5 h. Another portion of aqueous NaNO_2_ (6M, 27 mL, 12.4 g in 27.0 mL water) was added at 0 °C. The reaction mixture was stirred at ambient temperature for 3 d. The reaction mixture was cooled to 0 °C and H_2_SO_4_ (5M, 45mL) was added, followed by NaNO_2_ (15.3 g in 27.0 mL water) slowly and stirred at ambient temperature for 2 d. The reaction mixture was concentrated under reduced pressure (70°C, 100-200 mbar). The resulting solution was treated with an aqueous NaOH solution (10 M) until neutralization. A mixture of MeOH/acetone (3:1, 400 mL) was added and passed through a pad of celite and repeated the process 7-10 times. Toluene was added (20 mL) for toluene-water azeotrope. The residue was dissolved in 60 mL of MeOH and 15-20 mL of trimethyl orthoformate (15.3g, 221.7 mmol, 1.55 equiv) was added. After that conc. H_2_SO_4_ was added until pH 1. The mixture was stirred at 60 °C for 1 h. Reaction was quenched by NaOMe at 0 °C. After filtration, solvent was evaporated, and the residue was purified by silica-gel column chromatography (10% Methanol-ethyl acetate) to obtain the (*S*)-2,3-dihydroxypropanoate **2** as a colorless liquid (10.1 g, 84.10 mmol, 59% yield); **^1^H-NMR (400 MHz, CDCl_3_):** δ 4.24 (t, *J* = 4.2,3.3 Hz, 1H), 4.1 (s, 2H), 3.81 (d, *J* = 3.1 Hz, 1H), 3.77 (d, *J* = 4.4 Hz, 1H), 3.72 (s, 3H); **^13^C‑NMR (100 MHz, CDCl_3_):** δ 173.6, 71.8, 64.1, 52.9.

**C. Procedure for acetonide protection of (*S*)-2,3-dihydroxypropanoate 2**

The acetonide protected methyl ester **3** was prepared using a literature procedure. In an oven dried 250 mL round bottom flask, **2** (8.7g, 72.75 mmol) was taken in 40 mL of dry CH_2_Cl_2_ and 2,2-dimethoxypropane (18mL, 145.5 mmol, 2.0 equiv) was added. The resulting solution was stirred at 0 °C for 5 min, following which 1.4g (7.2 mmol, 0.1 equiv) of *p*-TSA was added and stirring was continued at 0 °C for 5 min. The reaction mixture was then stirred at 30-35 °C for 12 h. The solvent was evaporated, and the residue was purified by vacuum distillation using 15 cm long Vigreux column (oil bath temperature 130-140 °C, 14 mbar pressure) to get acetonide **3** as a yellow liquid (8.3 g, 51.8 mmol, 71% yield); **^1^H-NMR (400 MHz, CDCl_3_):** δ 4.53 (dd, *J* = 7.1, 5.4 Hz, 1H), 4.17 (dd, *J* = 8.5, 7.4 Hz, 1H), 4.04 (dd, *J* = 8.6, 5.2 Hz, 1H), 3.7 (s, 3H), 1.43 (s, 3H), 1.34 (s, 3H); **^13^C‑NMR (100 MHz, CDCl_3_):** δ 171.7, 111.4, 74.1, 67.3, 52.4, 26.0, 25.6.

**D. Procedure for the conversion of methyl ester to *N*,*N*-dimethyl amide**

The amide **4** was prepared using a modified literature procedure. The acetonide protected methyl ester **3** (3.3 g, 20.6 mmol) was dissolved in 15 mL ethanol and cooled to 0 °C. Dimethyl amine was added in portion over two hours (5.0 equiv × 2). After stirring for 36 h, the reaction mixture was concentrated in vacuo and the residue was purified by column chromatography (50-60% ethyl acetate in petroleum ether) to obtain the amide **4** as a pale yellow liquid (2.5 g, 14.43 mmol, 70% yield); **^1^H-NMR (400 MHz, CDCl_3_):** δ 4.47 (t, *J* = 6.6 Hz, 1H), 4.08 (dd, *J* = 8.3, 6.4 Hz, 1H), 3.87 (dd, *J* = 8.1, 6.9 Hz, 1H), 2.88 (s, 3H), 2.7 (s, 3H), 1.15 (s, 6H); **^13^C‑NMR (100 MHz, CDCl_3_):** δ 169.1, 111.6, 73.6, 66.5, 37.0, 35.9, 26.0, 25.9.

**E. Procedure for the conversion of amide to isopropenyl ketone**

The isopropenyl ketone **5** was prepared using a modified literature procedure. In an oven dried 25 mL round bottom flask, the amide **4** was taken (1.0 g, 5.7 mmol) under argon along with dry THF. The resulting solution was cooled to 0 °C. Freshly prepared isopropenylmagnesium bromide was the added dropwise to the solution of **4** at 0 °C. The reaction mixture was stirred for 1 h at rt and then quenched with aqueous NH_4_Cl solution. The organic layer was extracted with diethyl ether. The combined organic layer was concentrated in vacuo and the residue was purified by column chromatography (7-10% ethyl acetate in petroleum ether) to obtain **5** as a pale yellow liquid (2.5 g, 14.43 mmol, 70% yield); **^1^H-NMR (400 MHz, CDCl_3_):** δ 6.00 (s, 1H), 5.89 (S, 1H), 5.05 (t, *J* = 6.7 Hz, 1H), 4.20 (t, *J* = 7.9, 1H), 4.05 (dd, *J* = 7.9, 6.5 Hz, 1H), 1.88 (s, 3H), 1.39 (d, *J* = 3.2 Hz, 6H); **^13^C‑NMR (100 MHz, CDCl_3_):** δ 197.5, 142.6, 126.6, 110.6, 76.7, 66.3, 25.7, 25.4, 17.7.

**F. Procedure for the ozonolysis of isopropenyl ketone 5**

The diketone **6** was prepared using a modified literature procedure. The ketone **5** (0.7 mmol) was taken in a 25 mL round bottom flask along with 5 mL dry MeOH, and the solution was cooled at –78 °C for 10 minutes. Then O_2_ was passed through this solution for 5 min followed by O_3_ (at 40% power) until the starting consumed (monitored by TLC). Me_2_S (0.5 mL) was then added, and the solution was stirred at –78 °C for 10 min, followed by 24 h 30 °C. The reaction mixture was concentrated under reduced pressure and diluted with 3 mL water. The organic layer was extracted with ethyl acetate and washed with water. The combined organic layer was concentrated in vacuo and the residue was purified by column chromatography (20% ethyl acetate in petroleum ether) to obtain acetonide-protected DPD {**6**} as a yellow oil (70.0 mg, 0.406 mmol, 58% yield); **^1^H-NMR (400 MHz, CDCl_3_):** δ 5.10 (dd, *J* = 7.9, 5.4 Hz, 1H), 4.33 (t, *J* = 8.5 Hz, 1H), 3.96 (dd, *J* = 8.9, 5.4 Hz, 1H), 2.36 (s, 3H), 1.44 (s, 3H), 1.39 (s, 3H); **^13^C‑NMR (100 MHz, CDCl_3_):** δ 198.1, 194.6, 111.3, 76.8, 66.0, 26.0, 25.3, 24.5.

**References**

1. Sarkar R, Mukherjee S. Iridium-catalyzed enantioselective olefinic C(sp(2))-H allylic alkylation. Chem Sci. 2021;12(8):3070-5.

2. Stecko S, Michalak M, Stodulski M, Mucha Ł, Parda K, Furman B, et al. Practical one-pot synthesis of protected L-glyceraldehyde derivatives. Synthesis. 2012;44(17):2695-8.
