# Supplementary material for: LuxS/AI-2 regulates phoP/phoQ by a non-canonical mechanism to enhance acid stress survival in Salmonella Typhimurium
Source: PLoS Pathog. 2026 May 28;22(5):e1014244. doi: 10.1371/journal.ppat.1014244 (PMC13218499; doi:10.1371/journal.ppat.1014244)
Supplement: S1 Table — (DOCX) [file ppat.1014244.s013.docx]

**Table S1**

All the Bacterial strains and plasmids used in this study are given in Table -

| Strains/Plasmids | Characteristics | Sources |
| --- | --- | --- |
| STM *enterica* serovar Typhimurium 14028s (STM WT) | No antibiotics | Gift from Prof. M. Hensel |
| STM ∆*luxS* | Chl^R^ | This study |
| STM ∆*lsrB* | Kan^R^ | This study |
| STM ∆*lsrK* | Chl^R^ | This study |
| STM ∆*lsrR* | Kan^R^ | This study |
| STM ∆*luxS* ∆*lsrR* | Chl^R^ Kan^R^ | This study |
| STM ∆*luxS:luxS* | Chl^R^, Amp^R^ (complemented in pQE60 plasmid) | This study |
| STM ∆*phoP* | Chl^R^ | Laboratory stock(1) |
| *Vibrio campbellii* ATTC BAA-1117 | No antibiotics | ATTC |
| pKD3 plasmid | Chl^R^ resistance cassette | Laboratory stock(2, 3) |
| pKD4 plasmid | Kan^R^ resistance cassette | Laboratory stock(2, 3) |
| pKD46 plasmid | Plasmid expressing λ-red recombinase system, Amp^R^ | Laboratory stock(2, 3) |
| pQE60 vector | Low copy number plasmid, Amp^R^ | Laboratory stock(2, 3) |
| pQE60-*phoP*-*phoQ* Complemented plasmid | Amp^R^ | This study |
| pBAD:pHuji | Amp^R^ | Add gene |
| pET28a(+) | Kan^R^ | Addgene |
| pET28a(+)- *lsrR* | Kan^R^ | This study |

1. Hariharan V, Chowdhury AR, Rao SS, Chakravortty D, Basu S. phoP maintains the environmental persistence and virulence of pathogenic bacteria in mechanically stressed desiccated droplets. iScience. 2023;26(5):106580.

2. Garai P, Lahiri A, Ghosh D, Chatterjee J, Chakravortty D. Peptide utilizing carbon starvation gene yjiY is required for flagella mediated infection caused by Salmonella. Microbiology. 2016;162(1):100-16.

3. Eswarappa SM, Panguluri KK, Hensel M, Chakravortty D. The yejABEF operon of Salmonella confers resistance to antimicrobial peptides and contributes to its virulence. Microbiology. 2008;154(Pt 2):666-78.
