# Supplementary material for: LuxS/AI-2 regulates phoP/phoQ by a non-canonical mechanism to enhance acid stress survival in Salmonella Typhimurium
Source: PLoS Pathog. 2026 May 28;22(5):e1014244. doi: 10.1371/journal.ppat.1014244 (PMC13218499; doi:10.1371/journal.ppat.1014244)
Supplement: S2 Table — (DOCX) [file ppat.1014244.s014.docx]

**Table S2**

**Media Composition**

| **M9 Minimal Media**: | 2mM Magnesium Sulphate, 0.1mM Calcium Chloride, 0.4% Glucose, 10X M9 salts [Di-Sodium hydrogen phosphate (64g in 1000ml), Potassium Di-hydrogen phosphate (15g in 1000ml), Sodium Chloride(2.5g in 1000ml), Ammonium Chloride(5g in 1000ml)] at a final concentration of 1X. |
| --- | --- |
| **F-Media:** | 8µM Magnesium Chloride, 38mM Glycerol, 0.1% Cas-amino acids, 10X F-media salts (50mM Potassium Chloride, 75mM Ammonium Sulphate, 5mM Potassium Sulphate, 10mM Potassium Di-hydrogen phosphate, 1M Bis-Tris) at final concentration of 1X. Final pH adjusted to 5. |
