# Supplementary material for: LuxS/AI-2 regulates phoP/phoQ by a non-canonical mechanism to enhance acid stress survival in Salmonella Typhimurium
Source: PLoS Pathog. 2026 May 28;22(5):e1014244. doi: 10.1371/journal.ppat.1014244 (PMC13218499; doi:10.1371/journal.ppat.1014244)
Supplement: S3 Table — (DOCX) [file ppat.1014244.s015.docx]

**Table S3**

**Primers used in this study:**

| Primers | | Sequence 5'…….3' |
| --- | --- | --- |
| *luxS* knockout forward primer | CGGAGGTGACTAAATGCCATTATTAGATAGCTTCGCAGTC**CATATGAATATCCTCCTTAG** | |
| *luxS* knockout reverse primer | CTGGAACCGCTTACAAATAAGACTAAATATGCAGTTCCTG**GTGTAGGCTGGAGCTGCTTC** | |
| *luxS* knockout confirmation forward primer | GCAAAACACGCCTGACCCAA | |
| *luxS* knockout confirmation reverse primer | CAATACACTCTGGCATCGTG | |
| *luxS* expression forward primer | GCTCCAGAATATGACGGGCA | |
| *luxS* expression reverse primer | CGCACCGGCTTTTACATGAG | |
| *luxS* cloning forward primer | **CGCGGATCC**ATCGGAGGTGACTAAATGCC | |
| *luxS* cloning reverse primer | **CCCAAGCTT**TGGAACCGCTTACAAATAAGAC | |
| *lsrR* knockout forward primer | CAAAGTAAAGCCAGGTTATGACAATGAGCGATAATACGTTGG**CATATGAATATCCTCCTTAG** | |
| *lsrR* knockout reverse primer | GAATTATTTTCCCTGCGGTTTTCTGATCGGTAACCAGTGC**GTGTAGGCTGGAGCTGCTTC** | |
| *lsrR* knockout confirmation forward primer | GTGGCGTTAATCACGGTTAT | |
| *lsrR* knockout confirmation reverse primer | TATTGAATTGAGGTAAGTGT | |
| *lsrR* expression forward primer | GAATATCGCCTACTGCGCCT | |
| *lsrR* expression reverse primer | AAATAGCGTGCGGGATGTGA | |
| *lsrB* knock out forward primer | ATGGCAAGACACAGCATTAAAATGATCGCCTTACTCACTGCATATGAATATCCTCCTTAG | |
| *lsrB* knock out reverse primer | TGAAAATGACACGCTCCGGCAATAACACAATGCCGTTACCGTGTAGGCTGGAGCTGCTTC | |
| *lsrB* knockout confirmation forward primer | CGTCACCAAATCCTTGAATG | |
| *lsrB* knockout confirmation reverse primer | CCACAGCCTTTTAATGCGAA | |
| *lsrB* expression forward primer | AGAGTTTGGCCTGTGGGATG | |
| *lsrB* expression reverse primer | GGTGAAACGGTGACTTTGCC | |
| *lsrK* knock out forward primer | TGGCTCGACTCTGTACCCATACTGAATCAGGACATTACCTCATATGAATATCCTCCTTAG | |
| *lsrK* knock out reverse primer | CGCTTTCCAGAGGGATGTCGTTAAGCCACCATCGACCAGGTGTAGGCTGGAGCTGCTTC | |
| *lsrK* knockout confirmation forward primer | CGGTAACTATATCAATGCAC | |
| *lsrK* knockout confirmation reverse primer | GCGGTATTCATGATTCTTCT | |
| *lsrK* expression forward primer | GTGAAATGTGACCGAGCAGC | |
| *lsrK* expression reverse primer | CTTTATGCTCAGCGGCGAAC | |
| *phoP* expression forward primer | GATCTCTCACGCCGGGAATT | |
| *phoP* expression reverse primer | TGACATCGTGCGGATACTGG | |
| *phoQ* expression reverse primer | GCAGCAAACGAAAGGTGGTT | |
| *phoQ* expression reverse primer | TTTGCTCGCCATTTTCTGCC | |
| *phoP/phoQ* cloning forward primer | CGCGGATCCGAGATGATGCGCGTACTGGTT | |
| *phoP/phoQ* cloning reverse primer | CCCAAGCTTTGGAAGAACGCACAGAAATGT | |
| *ssrA* expression forward primer | ACCGCCCATCATTTTAGCCA | |
| *ssrA* expression reverse primer | AACCGGAGGGATACGTCTGA | |
| *ssrB* expression forward primer | CGGTGTGTTTCGACGGTTTT | |
| *ssrB* expression reverse primer | ACGCTGACACGACCAATCAT | |
| *ssaV* expression forward primer | CGCCGCAAAAAGTCTGTGGT | |
| *ssaV* expression reverse primer | GGGACGCCGGTATCCTCAAA | |
| *spiC* expression forward primer | ACCTAAGCCTTGTCTTGCCT | |
| *spiC* expression reverse primer | CCATCCGCTGTGAGCTGTAT | |
| *cadA* expression forward primer | CTGAACCTGCGCGTCAAAAA | |
| *cadA* expression reverse primer | TTTCGGCAGCACTTCAAACG | |
| *cadB* expression forward primer | TTGGCCTATTCCTGGTGCTG | |
| *cadB* expression reverse primer | GCGTTTCGGGTTTTTCACCA | |
| *cadC* expression forward primer | TCAATCAGCGCCACTATCGG | |
| *cadC* expression reverse primer | GATTAGGCCAGGGCTGTGTT | |
| *lsrR* cloning forward primer | CATGCCATGGACAATGAGCGATAATACGTTG | |
| *lsrR* cloning reverse primer | CCGCTCGAGTTTTTCAATAATTTGAATTATTTTCCCTGC | |
| Y25A *lsrR* SDM forward primer | CGTATTGCCTGGTTCGCCTATCACGATGG | |
| Y25A *lsrR* SDM reverse primer | CCATCGTGATAGGCGAACCAGGCAATACG | |
| R43A lsrR SDM forward primer | CTGGGGCTAACCGCGCTAAAGGTTTCTCG | |
| R43A lsrR SDM reverse primer | CGAGAAACCTTTAGCGCGGTTAGCCCCAG | |
| *lsr* promoter sequence | GACCAAATAACTACTACCGTTTTGAACAATTTCTTTTTCAAAAAACATTTGTTCAGTCCCGTCAGTCAACATTGAGGGAGCGGAGGCAAC | |
| *phoP* promoter sequence | AAGAGGGTGACTATTTGTCTGGTTTATTAACTGTTTATCCCCAAAGCACCATAATCAACGCTAGACTGTTCTTATTGTTAACACAAGGGA | |
| 16S rRNA forward primer | CGCTTCTCTTTGTATGCGCC | |
| 16S rRNA reverse primer | TCGTCAGCTCGTGTTGTGAA | |
