# Supplementary material for: LuxS/AI-2 regulates phoP/phoQ by a non-canonical mechanism to enhance acid stress survival in Salmonella Typhimurium
Source: PLoS Pathog. 2026 May 28;22(5):e1014244. doi: 10.1371/journal.ppat.1014244 (PMC13218499; doi:10.1371/journal.ppat.1014244)
Supplement: Attachment — Submitted filename: 4) Response to Reviewers.docx [file ppat.1014244.s016.docx]

**Response to Reviewer’s comments for** **Manuscript Number: PPATHOGENS-D-25-02450**

**The quorum-sensing lexicon of *Salmonella* ameliorates acid stress in the host by a non-canonical mechanism**

All changes made are highlighted in yellow in the revised manuscript, and the suggested text has been removed.

**Additional Editor Comments :**

Dear Dr. Chakravortty,

Thank you very much for submitting your manuscript "The quorum-sensing lexicon of Salmonella ameliorates acid stress in the host by a non-canonical mechanism" (PPATHOGENS-D-25-02450) for review by PLOS Pathogens. Your manuscript was fully evaluated at the editorial level and by three independent peer reviewers. The reviewers appreciated the attention to an important problem, but raised some substantial concerns about the manuscript as it currently stands. These issues must be addressed before we would be willing to consider a revised version of your study. We cannot, of course, promise publication at this time. We therefore ask you to modify the manuscript according to the review recommendations before we can consider your manuscript for acceptance. Your revisions should address the specific points made by each reviewer.

I am returning your manuscript with three reviews. The reviewers all agree that this work is new and interesting information for the field, but requires clarifications on the rationale and the way some some experiments were performed, analyzed and presented, and substantial language editing is needed to improve clarity and readability. After reading the reviews and looking at the manuscript, I recommend Major Revision based on the critiques from the more critical reviews. I am sorry I cannot be more positive at the moment, however we are looking forward to receiving your revision. With a a lot of work, the manuscript will be suitable for a resubmission, if you so wish to do so.

**Respected Editor,**

**Thank you very much for your detailed evaluation of our manuscript entitled “The quorum-sensing lexicon of Salmonella ameliorates acid stress in the host by a non-canonical mechanism” (PPATHOGENS-D-25-02450).**

**We sincerely appreciate the time and effort invested by you and the three reviewers in carefully assessing our work. We are encouraged by the reviewers’ recognition that the study addresses an important and novel aspect of the field. We also acknowledge the substantial concerns raised regarding experimental rationale, data analysis, presentation, and language clarity.**

**We fully understand the recommendation for a major revision and appreciate the constructive nature of the feedback. We are committed to thoroughly addressing each point raised by the reviewers. In the revised manuscript, we will clarify the experimental rationale, improve data analysis and presentation where require, incorporate additional methodological details, and undertake substantial language editing to enhance clarity and readability.**

**Thank you again for the opportunity to revise and resubmit our work. We look forward to submitting a carefully revised version of the manuscript that addresses all reviewer and editorial comments in detail.**

**Journal Requirements:**

1) Please provide an Author Summary. This should appear in your manuscript between the Abstract (if applicable) and the Introduction, and should be 150-200 words long. The aim should be to make your findings accessible to a wide audience that includes both scientists and non-scientists. Sample summaries can be found on our website under Submission Guidelines:
<https://journals.plos.org/plospathogens/s/submission-guidelines#loc-parts-of-a-submission>

**We have now included an Author Summary in the revised manuscript.**

2) We do not publish any copyright or trademark symbols that usually accompany proprietary names, eg ©,  ®, or TM  (e.g. next to drug or reagent names). Therefore please remove all instances of trademark/copyright symbols throughout the text, including:
- TM on page: 32.

**Thank you so much for the clarification. We have removed all trademark and copyright symbols throughout the manuscript, including the TM symbol on page 32, in accordance with the journal’s guidelines.**

3) We notice that your supplementary Tables are included in the manuscript file. Please remove them and upload them with the file type 'Supporting Information'. Please ensure that each Supporting Information file has a legend listed in the manuscript after the references list.

**We apologize for the oversight. The supplementary Tables have now been removed from the main manuscript and added to the Supporting Information.**

4) Some material included in your submission may be copyrighted. According to PLOSu2019s copyright policy, authors who use figures or other material (e.g., graphics, clipart, maps) from another author or copyright holder must demonstrate or obtain permission to publish this material under the Creative Commons Attribution 4.0 International (CC BY 4.0) License used by PLOS journals. Please closely review the details of PLOSu2019s copyright requirements here: PLOS Licenses and Copyright. If you need to request permissions from a copyright holder, you may use PLOS's Copyright Content Permission form.
Please respond directly to this email and provide any known details concerning your material's license terms and permissions required for reuse, even if you have not yet obtained copyright permissions or are unsure of your material's copyright compatibility. Once you have responded and addressed all other outstanding technical requirements, you may resubmit your manuscript within Editorial Manager. 
Potential Copyright Issues:
i) Figures 6A, 6G, 6O, and S9A. Please confirm whether you drew the images / clip-art within the figure panels by hand. If you did not draw the images, please provide (a) a link to the source of the images or icons and their license / terms of use; or (b) written permission from the copyright holder to publish the images or icons under our CC BY 4.0 license. Alternatively, you may replace the images with open source alternatives. See these open source resources you may use to replace images / clip-art:
- [https://commons.wikimedia.org](https://commons.wikimedia.org/)
- <https://openclipart.org/>.

**We apologize for any confusion. All images and schematic elements included in Figures 6A, 6G, 6O, and S9A were created by the authors using Microsoft PowerPoint and were not taken from any external sources. No third-party images, icons, or clip-art were used. Therefore, no additional copyright permissions are required.**

Note: If the figure is created through BioRender. Please state this in the figure legend. Please also confirm that you hold a Premium account and provide a pdf copy of the CC BY 4.0 Licence as provided by BioRender. For instructions on how to generate a CC BY 4.0 license for your figure, please see the guidelines here: <https://help.biorender.com/hc/en-gb/articles/21282341238045-Publishing-in-open-access-resources>. 
If you are using the free assets from BioRender, we are unable to publish these images as they are licenced under a stricter licence than CC BY 4.0. In this case we ask you to remove the BioRender images and replace them with open source alternatives.
See these open source resources you may use to replace images / clip-art:
- <https://bioart.niaid.nih.gov/> 
- <https://bioicons.com/>
- <https://healthicons.org/> 
- <https://scidraw.io/>
- <https://reactome.org/icon-lib>
- <https://www.phylopic.org/images> 
- <https://journals.plos.org/plosbiology/article?id=10.1371/journal.pbio.3002395>

**We apologize for any confusion. The relevant schematic figures (Graphical abstract) were created using BioRender. We confirm that we hold a BioRender Premium account, which allows publication under the CC BY 4.0 license. This information has now been stated explicitly in the corresponding figure legends, Created in BioRender. Chakravortty, D. (2026) w2ra69x https://BioRender.com and a PDF copy of the CC BY 4.0 license provided by BioRender has been uploaded as requested.**

5) We note that your Data Availability Statement is currently as follows: "The data is in the manuscript.". Please confirm at this time whether or not your submission contains all raw data required to replicate the results of your study. Authors must share the “minimal data set” for their submission. PLOS defines the minimal data set to consist of the data required to replicate all study findings reported in the article, as well as related metadata and methods (<https://journals.plos.org/plosone/s/data-availability#loc-minimal-data-set-definition>).
For example, authors should submit the following data: 
1) The values behind the means, standard deviations and other measures reported;
2) The values used to build graphs;
3) The points extracted from images for analysis..
Authors do not need to submit their entire data set if only a portion of the data was used in the reported study.
If your submission does not contain these data, please either upload them as Supporting Information files or deposit them to a stable, public repository and provide us with the relevant URLs, DOIs, or accession numbers. For a list of recommended repositories, please see <https://journals.plos.org/plosone/s/recommended-repositories>. 
If there are ethical or legal restrictions on sharing a de-identified data set, please explain them in detail (e.g., data contain potentially sensitive information, data are owned by a third-party organization, etc.) and who has imposed them (e.g., an ethics committee). Please also provide contact information for a data access committee, ethics committee, or other institutional body to which data requests may be sent. If data are owned by a third party, please indicate how others may request data access.

**We confirm that the submission contains all data required to replicate the results of this study. The minimal data set, including the values underlying all graphs, summary statistics, and image-derived quantifications, is provided within the manuscript and its Supporting Information files. Accordingly, the Data Availability Statement has been revised to state: “All relevant data are within the manuscript and its Supporting Information files.”**

6) Please amend your detailed Financial Disclosure statement. This is published with the article. It must therefore be completed in full sentences and contain the exact wording you wish to be published.
1) State what role the funders took in the study. If the funders had no role in your study, please state: "The funders had no role in study design, data collection and analysis, decision to publish, or preparation of the manuscript."

**Thank you for the clarification. We have added the following statement to the manuscript: “The funders had no role in study design, data collection and analysis, decision to publish, or preparation of the manuscript.”**

**Reviewers' Comments:**

Reviewer's Responses to Questions

**Part I - Summary**
Please use this section to discuss strengths/weaknesses of study, novelty/significance, general execution and scholarship.

Reviewer #1: This work investigates the importance of the Salmonella lsr quorum-sensing system of the ability of this pathogen to survive in macrophages and under acidic conditions. It demonstrates that mutants unable to produce (luxS), transport (lsrB), or phosphorylate (lsrK) the AI-2 signal, but not the repressor of the regulon (lsrR), are defective in these functions. The work implicates the PhoQ/PhoP regulator as the site of action of LsrR, demonstrating binding to the phoP promoter region that is absent in an LsrR mutant with two amino acid changes. In vivo studies using a mouse model of oral or peritoneal infection support the physiological importance of this control. Overall, the work produces new and interesting information, with some questions about techniques used and their interpretation.

**We sincerely thank the reviewer for the thorough evaluation of our manuscript and for the positive assessment of the novelty and physiological relevance of our work. We would be happy to address the questions regarding the experimental techniques used and their interpretation. Accordingly, we have clarified the methodological details, strengthened the description of controls, and expanded the discussion to ensure that the data and their interpretation are clearly and accurately presented in the revised manuscript.**

Reviewer #2: In their research, the authors examine the non-canonical quorum-sensing mechanism in Salmonella. The paper is clearly written and well organized, systematically explaining the initial thesis and demonstrating the proposed mechanism.

**We thank the reviewer for the positive and encouraging evaluation of our manuscript. We appreciate the reviewer’s recognition that the study is clearly written, well organized, and systematically presents both the underlying hypothesis and the proposed non-canonical quorum-sensing mechanism in Salmonella. We are grateful for these constructive comments, which reinforce the clarity and structure of our work.**

My questions and concerns are listed below:
Introduction:
The introduction section needs some reorganization; too many repetitive sentences make it difficult to read and a bit too long. Overall, it provides the necessary background information, but in a scattered manner.

**We thank the reviewer for this constructive suggestion. We agree that the Introduction contained repetitive sentences and could be improved for clarity and flow. Accordingly, we have reorganized the introduction to reduce redundancy, improve logical progression, and enhance readability. The revised version now presents the necessary background information more concisely and coherently.**

Results:
The justification and explanation of each part of the results section are too elaborate. Some of it should be placed in either the introduction or the discussion section. This makes the results section too long, and some information is repeated several times throughout the manuscript.

**We thank the reviewer for this helpful comment. We agree that portions of the Results section contain overly detailed justification and explanation, leading to unnecessary length and repetition. In response, we have streamlined the Results section by focusing it more strictly on the presentation of experimental findings. Explanatory and interpretative content has been relocated to the Introduction and Discussion sections, and repeated information has been removed throughout the manuscript. These revisions have improved the clarity, conciseness, and overall structure of the manuscript.**

Line 234: “Our study shows that environmental acidification is the critical physiological signal that upregulates AI-2 production by Salmonella (Fig. 2G).”
I am not sure how this figure is showing the above-mentioned observation. Please clarify. Upregulation compared to what exactly?

**We thank the reviewer for pointing out this lack of clarity and sincerely apologise for the confusion caused by the original statement. In Figure 2G, AI-2 production under acidic F-media conditions was compared to AI-2 production during growth in LB medium (Figure 1A, Now revised S1A). The LB-grown bacteria exhibited comparatively lower AI-2–dependent luminescence than bacteria grown under acidic conditions, which led to our conclusion that environmental acidification enhances AI-2 production. To avoid ambiguity, we have rephrased the statement in the revised manuscript to clearly specify the comparison and have clarified this point in the results section.**

Figures should be corrected (graphical point of view: figure alignment).

**We thank the reviewer for this observation. Accordingly, we have corrected the figure alignment and adjusted the layout to ensure visual consistency and clarity throughout the manuscript.**

Discussion

In the discussion section, the authors extensively cover microbiome composition; however, their focus is primarily on Salmonella mechanisms of communication and QS, with no direct experiments linked to or correlated with the microbiome study. Therefore, I recommend shortening this section of the discussion.
As a summary, I have to admit that I read this paper with a large dose of satisfaction. It is a well-planned and excellently executed study, which brings new data and knowledge to the field. It should also be of interest to the general public, as it addresses urgent topics in bacterial infection, bacterial communication, and potential antimicrobial strategies.

**We thank the reviewer for their careful evaluation of the discussion section and for their constructive suggestions. We agree that the microbiome-related content was overly extensive in relation to the experimental focus of this study, which centres on Salmonella quorum sensing and communication mechanisms. Accordingly, we have shortened and refined the discussion by reducing the microbiome-related content and limiting it to contextually relevant points, thereby improving focus and coherence.**

**We are grateful for the reviewer’s very positive overall assessment of our work. We sincerely appreciate the recognition of the study as well-planned and well-executed, and for highlighting its contribution to advancing knowledge in bacterial infections, quorum sensing, and antimicrobial strategies.**

Reviewer #3: The authors of the paper ‘The Salmonella quorum sensing lexicon attenuates acid stress in the host through a non-canonical mechanism’ demonstrate the role of the AI-2 autoinducer-dependent quorum sensing system in Salmonella's response to acid stress via the two-component PhoP/PhoQ system and subsequent regulation of SPI-2 and acid tolerance response genes. The phenotypic impact on the intracellular survival of Salmonella in macrophages and in vivo in a mouse model is also described. A great deal of work is presented. However, the paper is difficult to read and the logic behind the sequence of results is not always clear, especially at the beginning. That is why I would like to recommend the publication of this work, but only after addressing the specific points below and undergoing a substantial rewriting process. I hope that my comments/suggestions will help the authors to improve their article.

**We sincerely thank the reviewer for the thorough evaluation of our manuscript and for the constructive comments. We appreciate the positive assessment of the scope of the work and its relevance in elucidating the role of the AI-2 dependent quorum-sensing system in Salmonella acid stress response, intracellular survival, and in vivo virulence.**

**We acknowledge the reviewer’s concern regarding the readability of the manuscript and the clarity of the logical flow, particularly in the early sections. In response, we have undertaken a substantial revision of the manuscript to improve clarity, coherence, and narrative structure.**

**Part II – Major Issues: Key Experiments Required for Acceptance**
Please use this section to detail the key new experiments or modifications of existing experiments that should be absolutely required to validate study conclusions.

Generally, there should be no more than 3 such required experiments or major modifications for a "Major Revision" recommendation. If more than 3 experiments are necessary to validate the study conclusions, then you are encouraged to recommend "Reject".

Reviewer #1: 1 .Fig. 1A and Fig. 2G show production of AI-2 during the growth cycle. Fig. 2G, however, does not show the growth of the cultures. An appropriate way to show these data and compare between them would be to use normalized luminescence (RLU/OD600). This would allow the reader to assess the differences in AI-2 production per bacterium.

**Thank you for this valuable suggestion. To address the reviewer’s concern regarding growth, we have now incorporated the growth curve of the STM WT strain directly in Fig. 2G. This allows readers to clearly assess AI-2 production in the context of bacterial growth while preserving a data representation that more faithfully reflects the luminescence measurements obtained in our assays.**

**Thank you for this insightful comment. We agree that normalization of AI-2 dependent luminescence to bacterial growth (RLU/OD600) can, in principle, facilitate comparison of AI-2 production on a per-cell basis. In our experimental setup, however, we observed that presenting the data as RLU/OD600 resulted in disproportionate variability and did not accurately reflect the underlying AI-2 production dynamics across the growth phases, thereby complicating data interpretation.**

2. Fig. 2A: The suggestion is that the luxS mutant cannot use AI-2 produced by the wild type in a macrophage. The more likely explanation is that the two strains did not reside in the same phagolysosome. This is mentioned in the Discussion but should be the primary conclusion here.

**We thank the reviewer for this important clarification. We agree that the data shown in Fig. 2A do not allow us to conclude that the luxS mutant is unable to respond to AI-2 produced by the wild-type strain within macrophages. As the reviewer correctly notes, a more plausible explanation is that the co-infecting strains may not localize to the same phagolysosomal compartment, thereby preventing effective AI-2 sharing.**

**Accordingly, we have revised both the results and discussion sections to reflect this interpretation. In the results, we now emphasise that co-infection with the wild-type strain does not rescue the intracellular survival defect of the *luxS* mutant, without implying direct failure of AI-2 utilisation. We explicitly state that the physical segregation of bacteria within distinct *Salmonella*-containing vacuoles (SCVs) likely limits intercellular AI-2 signalling, which is consistent with known heterogeneity in macrophage phagosome maturation and bacterial compartmentalisation.**

3. Fig. 2G-J shows the effects of pH on AI-2 production and its consequences on bacterial survival using F-medium adjusted to various pH levels. These results, however, are compared to those of Fig. 1, for which LB medium was used. The experiments should all use the same medium with pH adjustments, as the two are very different from each other; either LB at low pH, or F-medium at neutral pH.

**We apologise for the confusion. To address the effect of pH independently of medium composition, we performed survival assays in both media with pH adjustments. The data shown in Fig. 2H–J were generated using PBS across a defined pH range. Complementary survival assays using LB medium adjusted to different pH values are provided in Supplementary Fig. S3D–G. Together, these experiments demonstrate that the observed effects of pH on AI-2–dependent survival are consistent across media, indicating that the conclusions are not medium-specific.**

4. Fig. 3A needs to be better labeled. It also might be of greater value if it were presented earlier in the manuscript.

**We thank the reviewer for this suggestion. Fig. 3A has been revised to improve labeling clarity, including clearer annotation and key components to enhance interpretability. In addition, we agree that this figure provides important contextual information and have therefore moved Fig. 3A earlier in Fig. 1A in the manuscript to better align with the logical flow of the results.**

5. Fig. 3BC tests expression of phoP in LB medium. Is not phoP poorly expressed under these conditions, requiring low pH, magnesium, and iron for induction? Using phoP-inducing conditions would be appropriate.

**We thank the reviewer for this important and insightful comment. The reviewer is correct that phoP expression is weak in standard LB medium and is optimally induced under acidic pH and low Mg²⁺/Fe²⁺ conditions. To directly address this point, we have repeated the experiment under phoP-inducing conditions using acidified LB medium, which robustly supports phoP activation. Moreover, phoP expression was independently examined in F-medium adjusted to acidic pH, and these results was presented in the Supplementary Figure S4E. Importantly, both inducing conditions yield consistent expression patterns, demonstrating that the conclusions drawn from Fig. 3BC (previously) are not influenced by non-inducing growth conditions but instead reflect a reproducible biological effect.**

**Fig. 3(A) *phoP* gene expression in STM WT, STM Δ*luxS*, STM Δ*lsrB*, and STM Δ*lsrK* *in vitro* conditions, LB medium (acidic pH 5)**

**Fig. 3(B) mRNA expression of *phoP in* STM Δ*luxS* upon treatment of STM WT spent medium (acidic pH 5).**

**S4(B) mRNA expression of *phoP* gene in STM WT (B)LB media (acidic pH5)**

6. Fig. 3G attempts to determine the cytoplasmic pH using the pHuji plasmid. These presumed pH values are, in fact, extrapolated from those obtained from culture of the bacteria in media with various pH values. Growth at a specific pH, however, does not equate to cytoplasmic pH, as bacteria possess extensive systems to buffer the cytoplasm. The approach may be helpful in some relative way to demonstrate acidification of the cytoplasm but is not correct as stated.

**We thank the reviewer for this important clarification. We agree that growth in media adjusted to a specific external pH does not directly equate to cytoplasmic pH, as bacteria possess robust buffering and homeostatic mechanisms to maintain intracellular pH. Accordingly, the pH values reported using the pHuji reporter should be interpreted as relative changes in cytoplasmic acidification rather than absolute intracellular pH measurements.**

**The pHuji-based approach was employed to compare relative cytoplasmic pH shifts across strains and conditions, and not to claim precise intracellular pH values. We have now revised the text to explicitly clarify this limitation, rephrasing the results to state that Fig. 3G reflects relative cytoplasmic acidification trends inferred from pHuji fluorescence calibration rather than direct cytoplasmic pH determination. Importantly, the comparative trends remain valid and support our conclusions regarding differential acid-stress responses between strains.**

7. Fig. 4F: The useful statistical comparison for the point mutants would be to the delta-luxR, delta-lsrR strain with the wild-type lsrR plasmid (purple bar).

**We thank the reviewer for this helpful suggestion. We agree that the most informative statistical comparison for the point mutants is relative to the *ΔluxS ΔlsrR* strain complemented with the wild-type lsrR plasmid (purple bar). Accordingly, we have now added these specific statistical comparisons to Fig. 4F. Inclusion of this analysis allows a more accurate assessment of the functional impact of the individual point mutations in lsrR relative to the fully complemented control and strengthens the interpretation of the data.**

8. The mice and macrophages used here are C57BL/6J, which is an nramp-negative strain with macrophages deficient in killing Salmonella. Would this have affected the results?

**We thank the reviewer for raising this important point. We acknowledge that C57BL/6J mice carry a nonfunctional Nramp1 allele and that macrophages derived from this background display reduced bactericidal activity against Salmonella. However, this genetic background is widely used and well established for studying intracellular survival mechanisms of Salmonella, as it allows robust bacterial persistence and thereby facilitates the dissection of host–pathogen interactions.**

**Importantly, all strains were compared within the same host and macrophage background, ensuring that the observed differences in intracellular survival reflect bacterial genotype-dependent effects rather than host genetic variability. The use of a Nramp1-deficient background, therefore, does not confound the conclusions, but instead provides a sensitive system to reveal differences in acid stress tolerance and quorum-sensing–dependent survival mechanisms.**

9. Line 327 and following: The AlphaFold3 results are written as if they are certain; the importance of only the Y25 and R43 residues are verified.

**We appreciate the reviewer’s comment and agree that AlphaFold3 predictions should not be interpreted as definitive evidence on their own. We have revised the text to clarify that AlphaFold3 was used as a hypothesis-generating approach to identify candidate DNA-contacting residues, rather than to draw firm conclusions. While the structural models suggested that multiple residues within the N-terminal domain (Y25, T31, Q32, S33, R43, L44, K45, S47, and R48) could potentially contribute to promoter interactions, Y25 and R43 were deliberately selected as representative residues from two distinct α-helices within the N-terminal domain, each showing the highest predicted interaction density with DNA in the AlphaFold3 models. These residues exhibited the strongest predicted contacts, lowest PAE values, and a robust co-variation signal from sequence covariance analysis. Their functional relevance was independently confirmed by EMSA and infection assays: single mutants (Y25A or R43A) showed progressively reduced promoter binding and regulatory activity, while the double mutant (Y25A/R43A) nearly abolished DNA binding and fully restored virulence phenotypes to knockout levels. Thus, although AlphaFold3 guided residue selection, our conclusions regarding Y25 and R43 are supported by convergent computational, biochemical, and functional evidence, rather than by structural prediction alone.**

Reviewer #2: In my opinion, this work addresses the studied subject with all the necessary experiments, and at this point, I do not recommend any major additional experiments. One aspect I was already considering was covered by the authors in the discussion section. It might be interesting to explore single-cell infection models and examine how co-infection occurs within a single macrophage. Furthermore, since the mutant does not survive in the infected macrophage and SPI-2 is not activated, I would like to see the macrophage polarization after infection, for example, M1/M2 phenotypes.

**We thank the reviewer for the very positive evaluation of our study and for recognizing that the work addresses the central questions with the necessary experimental depth. We also appreciate the thoughtful suggestions regarding single-cell infection models and macrophage polarization. We agree that single-cell approaches examining co-infection within individual macrophages would be an interesting and valuable future direction; however, such analyses are beyond the scope of the current study and are now mentioned as a potential avenue for further investigation.**

**With respect to macrophage polarization, while we have not explicitly classified macrophages into M1/M2 phenotypes, we have assessed host inflammatory responses by quantifying cytokine expression following infection. As shown in Fig., infection with quorum-sensing defective mutants leads to altered expression of key pro- and anti-inflammatory cytokines, including TNF-α and IL-10. We observed that STM WT was more efficient in polarising toward M2 macrophages. These data provide functional insight into macrophage activation states and suggest differential immune modulation by the mutants, consistent with their impaired intracellular survival and reduced SPI-2 activation. We have clarified this point in the Discussion to better contextualise these findings within macrophage polarisation.**

Reviewer #3: • All along the results:
- the results are often summarised in a single sentence for each figure or supplementary figure subsection. The authors' reasoning is not explained, and it therefore requires considerable effort from the reader to understand why certain experiments were carried out and the results obtained. For example, in the first paragraph, the authors want to demonstrate that AI2 is required for the survival of Salmonella in macrophages (Figure 1E). However, the authors show in Figure 1D that phagocytosis of the luxS, B or K mutants is significantly increased compared to the wt strain, and they give the same value to these two results. It would have been clearer to write that they observed impaired survival of these mutants in macrophages (Figure 1E) and that this reduced survival is not related to an impaired phagocytosis (Figure 1D). By contrast, as expected, the lsrR mutant and the complemented luxS mutant behaved as the wt. It would thus be desirable to expand on the results a little more to explain them better, along with the associated reasoning in most paragraph of results.

**We thank the reviewer for this helpful comment and agree that the results section, in its original form, was too concise and did not sufficiently explain the rationale for certain experiments. To address this concern, we have expanded the results text throughout the manuscript to better articulate the reasoning underlying each experiment and to more clearly link related results. In particular, we have revised the first results paragraph to explicitly distinguish between bacterial phagocytosis and intracellular survival in macrophages. Specifically, we now clarify that although the *luxS***, ***lsrB***, **and *lsrK* mutants exhibit increased phagocytosis compared to the wild-type strain (Figure 1D). We explicitly state that this reduced survival is therefore not attributable to impaired phagocytosis, but rather reflects a defect in post-uptake survival within macrophages. In contrast, as expected, both the *lsrR* mutant and the complemented *luxS* strain behave similarly to the wild type in terms of both uptake and survival. Similar explanatory text has been added to other results paragraphs to provide a clearer interpretation of the data and to guide the reader through the authors’ experimental reasoning.**

- Statistical analyses: except for the in vivo experiments, parametric tests have been performed all along the paper. This assumes that the data are normally distributed. As most of the time n<10, to my opinion, statistical non-parametric tests should have been performed as the Gaussian distribution cannot be insured with this number of data.

**We thank the reviewer for this thoughtful comment regarding the choice of statistical tests. We acknowledge that parametric tests formally assume normal data distribution. However, we chose parametric analyses for most experiments for several important reasons.**

**Our data represent controlled biological experiments rather than heterogeneous population-level measurements. Replicates were generated under identical experimental conditions, and variability across experiments was low and symmetric, supporting the appropriateness of parametric approaches.**

**While non-parametric tests are useful when data are clearly non-normal or highly variable, they are also known to be more conservative and less powerful for N=3. In our case, applying nonparametric tests substantially reduced statistical power and obscured biologically meaningful, reproducible differences consistently observed across independent experiments. Given that the same conclusions were reproducible across repeats, we believe that parametric tests provide a more accurate and informative statistical assessment of the data.**

**However, for *in vivo* experiments, where variability is inherently higher, we specifically used non-parametric tests, reflecting a deliberate, context-dependent choice of statistical methodology rather than a uniform application of a single approach.**

**We surveyed several key studies pertaining to this area of research and observed that these studies have represented the data with N=3 or 4 and performed statistical analysis using parametric t-Tests and ANOVA for all in vitro experiments carried out under similar experimental conditions. We also selected the same, as they are standard and robust for controlled cell-culture and other in-vitro experiments. Some of the key research articles are listed below:**

[**https://doi.org/10.1371/journal.pbio.3002731**](https://doi.org/10.1371/journal.pbio.3002731)

**https://doi.org/10.1038/s41586-025-09415-y**

[**https://doi.org/10.1016/j.redox.2024.103151**](https://doi.org/10.1016/j.redox.2024.103151)

[**https://doi.org/10.1371/journal.ppat.1013453**](https://doi.org/10.1371/journal.ppat.1013453)

**Taken together, we believe that the use of parametric statistical tests in this study is consistent with field standards and appropriate for the experimental design and data structure presented.**

**However, we would be happy to make further changes to the biostatistical test to fully address the reviewer's point.**

- RT-qPCR results: Lines 782-785: no control of the absence of DNA on DNase treated samples is mentioned. Is this an oversight? This step is crucial to ensure a right quantification of RNA. Moreover, only the 16S rRNA gene was used as “housekeeping gene”. The recommendation is to use at least two. Figure 1F and 1G and other graphs showing RT-qPCR results: If the results are expressed as “fold expression of the gene of interest /16S”, why is the ratio obtained always equal to 1 for all genes tested at 2h pi? Moreover, concerning results in Figure S3A,S3B, S4A to S4C, how do you explain that in F acidic medium or in RAW macrophages you obtained the same fold expression/16S as in LB medium (figure 1B and 1C) if AI-2 production is up-regulated at acidic pH. Figure 5A and B: Results obtained on ssrA and ssrB are surprising as these two open reading frames are co-transcribed.

**RT-qPCR controls and normalisation**

**We thank the reviewer for raising this important methodological point. As mentioned in the methods (RNA isolation and RT PCR), all RNA samples were first treated with the DNase enzyme. To confirm DNA contamination, we performed PCR with 16S primers. No amplification was detected in these controls, indicating efficient DNase treatment and validating the RNA-specific quantification. We acknowledge that this control was not explicitly mentioned in the original methods section and have now clarified this point in the revised manuscript**.

**Regarding normalisation, we agree that using more than one housekeeping gene is generally recommended. In this study, 16S rRNA was selected because its expression was experimentally verified to be stable across all tested conditions. We followed the protocol for our previously published laboratory research paper and established methods from the literature as well.**

[**doi.org/10.1371/journal.ppat.1010708**](https://doi.org/10.1371/journal.ppat.1010708)

**doi.org/10.1371/journal. ppat.1013453**

**doi.org/10.1016/j.redox.2024.103151**

**doi.org/10.1016/j.chom.2023.12.001**

**Fold expression equal to 1 at 2 h post-infection (Figures 1F, 1G, and related RT-qPCR graphs)**
**The fold expression value of 1 observed at 2 h post-infection reflects the normalization strategy used for RT-qPCR analysis. For each gene, expression levels at subsequent time points were calculated relative to the 2 h post-infection condition, which was used as the calibrator. We have now clarified this normalization approach explicitly in the methods section and in the figure legends to avoid any ambiguity. Fold-differences in gene transcription were calculated as 2^−ΔΔCt^**

**Comparable fold expression in acidic medium, macrophages, and LB (Figures S3A, S3B, S4A–S4C)**
**Although AI-2 production is up-regulated under acidic conditions, this increase does not necessarily lead to proportional changes in absolute transcriptional levels across different experimental conditions. In our RT-qPCR analysis, gene expression was calculated relative to the initial time point specific to each condition. In LB and acidic F medium, fold expression values were normalized to the respective 3 h time point, whereas in RAW macrophages, expression levels were calculated relative to 2 h post-infection, which served as the calibrator.**

**As a result, fold change values reflect relative temporal changes within each condition, rather than direct comparisons of absolute transcript abundance between LB, acidic medium, and intracellular macrophage environments. The apparently similar fold expression across conditions therefore reflects comparable regulation relative to their own baseline time points, despite differences in AI-2 production. We have clarified this normalization strategy in the methods section to better explain this point.**

**Expression of *ssrA* and *ssrB* (Figure 5A and 5B)**

**We agree with the reviewer that the differences observed in *ssrA* and *ssrB* expression are unexpected given that these genes are co-transcribed within the same operon. However, differential transcript abundance within polycistronic operons has been reported previously and may arise from differences in mRNA stability, processing, or regulatory feedback mechanisms. Liew et al. (eLife, 2019) showed that when *Salmonella* enters acidic compartments within host cells, low pH alters the shape of the SsrB regulator, enabling it to bind DNA and activate SPI-2 virulence genes required for intracellular survival.**

Andrew Tze Fui Liew et.al 2019, eLife

• Macrophage infections: The results are expressed as phagocytosis percentages and proliferation rates. Phagocytosis rates appear to be very low, less than 2%, while proliferation rates are very high (40 to 50) compared to the literature. Was there a transcription error?

**We thank the reviewer for raising this point. We confirm that there is no transcription error** **in the reported phagocytosis percentages or proliferation rates. The relatively low phagocytosis percentages (<2%) reflect the stringent infection conditions used in this study, including the multiplicity of infection, infection time, and the post-infection gentamicin treatment, which were designed to minimize extracellular carryover and to ensure that only truly internalized bacteria were quantified. Similar low uptake values have been reported in macrophage infection assays performed under comparable experimental conditions. Conversely, the high proliferation values (40–50) represent fold increases relative to the initial intracellular bacterial load, rather than absolute bacterial numbers. This is different from phagocytosis, where the percentage is from the initial inoculum of bacteria provided during infection to the cells.**

**Same phenotype we observed in our previous lab study-**

[**doi- 10.1371/journal.ppat.1010708**](https://doi.org/10.1371/journal.ppat.1010708)

**doi- 10.1016/j.redox.2024.103151**

• Figure 3: Complementation of at least the luxS mutant would improve the results presented in this figure.

**We thank the reviewer for this valuable suggestion. We agree that complementation strengthens the conclusions. Accordingly, we have now complemented the *luxS* mutant included the corresponding data. The complemented strain data have been incorporated into Figure 3A, 3B, and 3E in the revised manuscript, with additional supporting results provided in the Supplementary figure.**

• Figure 4E, 4F and figure 6: complementation of the luxS mutant with the phoP gene under a non-native promoter would confirm the results.

**We agree with the reviewer. To address this point, we have performed the suggested complementation experiment by expressing *phoP* under a non-native promoter in the *luxS* mutant background. The results confirm our conclusions and are now included in the revised manuscript as Supplementary Figure**, **which supports the data shown in Figures 4E, 4F, and 6.**

**Supporting data for Figures 4E, 4F- revised and shown in Supplementary Figure S8**

**Supporting data for Figures 6- Revised as Supplementary Figure S11**

• Thijs et al. (https://doi.org/10.1038/cr.2010.104) results should be discussed as they did not found phoP as part of the LsrR regulon

**We thank the reviewer for pointing out the study by Thijs et al. (Cell Research, 2010; DOI: 10.1038/cr.2010.104), which reported that *phoP* was not identified as part of the LsrR regulon. We agree that this observation is important and have now discussed it explicitly in the manuscript. In the study by Thijs et al., the LsrR regulon was defined using genome-wide transcriptional profiling to identify direct regulatory targets mediated by canonical double-stranded DNA promoter binding. In contrast, our study demonstrates that LsrR interacts with the *phoP* promoter to its single-stranded DNA. This distinction provides a plausible explanation for why *phoP* was not identified as an LsrR-regulated gene in the transcriptomic analysis performed by Thijs et al. We have clarified this point in the discussion to emphasise that our findings do not contradict previous work but instead reveal a non-canonical mode of LsrR DNA interaction.**

**Part III – Minor Issues: Editorial and Data Presentation Modifications**
Please use this section for editorial suggestions as well as relatively minor modifications of existing data that would enhance clarity.

Reviewer #1: 10. Some abbreviations are overused, making the manuscript difficult to read. Most notable is STM. As Salmonella Typhimurium is the only organism used here, that can be omitted throughout.

**We thank the reviewer for this suggestion. We agree that the repeated use of abbreviations can reduce readability. As Salmonella Typhimurium is the only organism used in this study, we have removed the abbreviation “STM” throughout the manuscript and replaced it with Salmonella Typhimurium or Salmonella, as appropriate. However, for the knockout strains, we retained the original nomenclature (e.g., STM *ΔluxS*).**

11. Line 147 and elsewhere: The term “luxS complemented STM delta-luxS” is confusing. Adding a hyphen (luxS-complemented) would help.

**We thank the reviewer for this helpful suggestion. We agree that the wording was confusing. We have revised the text throughout the manuscript to use the term “*luxS*-complemented STM *ΔluxS*,” adding a hyphen.**

12. Line 192 and elsewhere: DPD is not defined.

**We thank the reviewer for pointing this out. We have now defined DPD at its first occurrence and also have incorporated additional information explaining the *luxS*/AI-2 signaling system in the introduction section, as suggested by Reviewer 3.**

Reviewer #2: The justification and explanation of each part of the results section are too elaborate. Some of it should be placed in either the introduction or the discussion section. This makes the results section too long, and some information is repeated several times throughout the manuscript.

Data presentation of the figures should be better organized, as right now the figures are not aligned properly.

**We thank the reviewer for this constructive comment. We have streamlined the results section by removing extensive justifications and explanatory text and relocating this material to the Introduction or Discussion sections, as appropriate. This revision reduces redundancy and improves the clarity and flow of the Results section. In addition, we have reorganized the figures to improve alignment and overall data presentation, ensuring that related panels are grouped logically and presented consistently throughout the manuscript.**

Reviewer #3: • I would recommend to modify the title to introduce Ai-2 and PhoP/Q two-component system

**We thank the reviewer for this valuable suggestion. In response, we have revised the title to explicitly introduce both the AI-2 signaling system and the PhoP/PhoQ two-component system. The new title now reads-**

**LuxS/AI-2 regulates *phoP/phoQ* by a non-canonical mechanism to enhance acid stress survival in *Salmonella* Typhimurium**

• Introduction The luxS/AI2 system should be presented in the introduction section to facilitate reading by scientists unfamiliar with this system. Figure 3A could be used to illustrate how the system works. Without this information, luxS (line 71), LsrR (lines 88 and 115), the lsr operon (line 117) are not understandable by people not working on the AI2/LuxS system.

**We sincerely apologise for the lack of clarity in the original version of the manuscript. As suggested by reviewers 1 and 3, we have revised to include a concise description of the LuxS/AI-2 signaling system to facilitate understanding by readers who are not familiar with this pathway. In addition, the schematic previously shown in Figure 3A has been better labeled and repositioned as Figure 1A, where it now illustrates the LuxS/AI-2 system and the organization of the *lsr* locus. These changes improve the clarity of references to LuxS, LsrR, and the lsr operon throughout the manuscript.**

• The lsr operon contains only the lsrA, D, C, B, F and G open-reading frames. Not lrsR and lsrK. Please write correctly all over the paper.

**We thank the reviewer for this important clarification. We agree that the *lsr* operon comprises only the *lsrA*, *lsrD*, *lsrC*, *lsrB*, *lsrF*, and *lsrG* open reading frames. The regulatory genes *lsrR* and *lsrK* are not part of the *lsr* operon and are transcribed independently. We have corrected the terminology throughout the manuscript to accurately reflect the genetic organization of the *lsr* locus, replacing all instances where *lsrR* and *lsrK* were incorrectly described as components of the *lsr* operon.**

• Please replace media by medium all along the manuscript

**We thank the reviewer for this suggestion. We have replaced the term “media” with “medium” throughout the manuscript.**

• First paragraph of the results lines 123 to 180 : This paragraph and figure 1 aim to demonstrate that LuxS/AI2 is required for Salmonella to survive in macrophages. Figures1A,B,C are therefore not required as these results concern bacteria grown in LB medium. I would suggest to remove them and to add figure S1D on figure 1.

**We thank the reviewer for this insightful suggestion. Figures 1A–C were initially included to establish the baseline behavior of the strains under standard growth conditions; however, we agree that these data are not essential for directly addressing the role of LuxS/AI-2 in macrophage survival. Accordingly, we have removed Figures 1A–C from Figure 1 and have incorporated Figure S1D into Figure 1H, as suggested. This revision sharpens the focus of the figure and the corresponding results section on the role of LuxS/AI-2 during macrophage infection.**

• Figure S1E and S1F could not be used to show a decreased ability to proliferate in macrophages. This assertion should be supported by quantification of intracellular Salmonella in several randomly selected fields and in at least three independent experiments.

**We thank the reviewer for this important comment. Figures S1E and S1F were included to qualitatively visualize the intracellular niche of *Salmonella* rather than to quantitatively assess intracellular proliferation. Fluorescence microscopy revealed that all strains localized within intact Salmonella-containing vacuoles. Based on these observations, we conclude that all strains are capable of residing within the SCV. We have revised the text accordingly to avoid over-interpretation of these images as evidence of altered intracellular proliferation.**

• Second paragraph of results, lines 181 to 225: In my opinion, these results, which are nevertheless interesting, do not contribute to demonstrating the role of AI-2 in the survival of Salmonella in acidic conditions. Moreover, I do not agree with the conclusion that the luxS mutant cannot acquire AI-2 from a wt strain when intra-vacuolar in macrophages after co-infection of the two strains. Indeed, to conclude this, the authors should provide evidence that wt and mutant strains are simultaneously present in the same vacuole. If not, the mutant could not acquire AI2, because it is not in the same vacuole as the wt and not because it is not able to do so. I would suggest to remove all this paragraph and figure 2A to 2F.

**We thank the reviewer for this careful evaluation. We agree that these results do not by themselves conclusively demonstrate a role for AI-2 in *Salmonella* survival under acidic conditions. We also acknowledge the limitation of the co-infection experiment, as it does not allow us to determine whether STM *ΔluxS* and STM WT are simultaneously present within the same host cells. As the observations reflect a global phenotype rather than single cell or single vacuole resolution, we cannot definitively conclude whether STM *ΔluxS* fails to acquire AI-2 due to an intrinsic inability to utilize AI-2 or because it does not share the same intracellular niche as the WT strain.**

**In response to this concern, and in line with the recommendation of Reviewer 1, we have now explicitly incorporated this limitation into the results section, where it was previously discussed only in the discussion. We have also revised the text to avoid over-interpretation of these findings. While we cannot exclude the possibility that STM *ΔluxS* is unable to utilize AI-2 even when infecting the same cell, potentially due to repression of the *lsr* operon this remains speculative and is now clearly stated as such.**

**However, we have retained the data presented in Figures 2A–F, as these experiments demonstrate that STM Δ*luxS* is capable of utilizing AI-2 under *in vitro* culture conditions and that inhibition of AI-2 signaling effectively blocks this response and its downstream consequences. While these data do not resolve AI-2 utilization during intracellular co-infection, they provide important mechanistic support for AI-2–dependent signaling under defined conditions. The text has been carefully revised to ensure that conclusions drawn from these experiments are limited to the in vitro context.**

• Third paragraph of the results: Figure 2G could be compared to the results in LB (Figure S1A) to demonstrate the up-regulation of AI-2 production at acidic pH. Perhaps, they can be merged?

**We thank the reviewer for this helpful suggestion. While a direct comparison between Figure 2G and Figure S1A could demonstrate differences in AI-2 production between LB and acidic conditions, we have chosen to keep these data in separate panels to maintain a clear focus on AI-2 regulation under acidic conditions, which is the primary emphasis of this study. Importantly, this rationale and the relevant comparison have now been explicitly explained in the revised manuscript.**

• Figure 3C: in which growth medium were the cultures done? Figure 3F, why did the luxS, lsrB and lsrK mutants better survive at pH3 when unadapted at pH 5 compared to adapted at pH5?

**We thank the reviewer for raising these important points. The experiments shown in Figure 3B and 3C were originally performed in neutral LB medium. However, in response to Reviewer 1’s suggestion, we have now repeated these experiments under acidic conditions. The revised experiments were carried out in acidic LB medium as well as in F medium. The data obtained in acidic LB medium are now presented in the main Figure 3A and 3B as they are directly relevant to the acid stress conditions emphasized in this study. For completeness, the results obtained in neutral LB and acidic F medium are now provided in the Supplementary Figure S3C and S3F.**

**We thank the reviewer for this very pertinent observation. We agree that the initially observed higher survival of the *luxS*, *lsrB*, and *lsrK* mutants at pH 3 under unadapted conditions compared to pH 5 adapted conditions was unexpected. To address this point, and in response to a major comment, we repeated the experiment using the *luxS-*complemented strains. In the revised dataset, the survival of the mutants is now comparable under both adapted and unadapted conditions, indicating that the previously observed difference was not a robust or reproducible phenotype.**

**We propose that the initial discrepancy may have resulted from altered stress-response dynamics in the absence of LuxS/AI-2 signaling, whereby pre-adaptation at pH 5 may transiently dysregulate acid tolerance pathways rather than effectively prime them. Such responses are known to be sensitive to growth conditions and strain background. Importantly, the inclusion of complemented strains in the revised experiments strengthens the conclusion that LuxS–AI-2–associated components do not confer a consistent survival advantage under these conditions.**

• Figure 3H: only two experiments were performed. It is therefore not possible to provide a standard deviation and to perform statistics on these data. At least 3 experiments would be better.

**We thank the reviewer for this important comment. We have now performed an additional independent experiment and incorporated the data into Figure. The figure has been updated accordingly (revised Figure 3G), and statistical analysis is now based on three independent experiments**

• Line 280: please “compared to the luxS, lsrB and lsrK mutants” after upon exposure to pH3.

**We thank the reviewer for this suggestion and have now added the comparison “compared to the *luxS*, *lsrB*, and *lsrK* mutants upon exposure to pH 3” in the revised manuscript.**

• Figure 4 legend: Line 374 please add respectively after promoters

**We thank the reviewer for this suggestion and have added “respectively” after “promoters” in the Figure 4 legend in the revised manuscript.**

• Lines 461 to 465 : how can you explain the reduced colonization of mice intraperitoneally inoculated with the luxS, lsrB and lsrK mutants

**We thank the reviewer for this important question. The reduced colonization observed following intraperitoneal inoculation with the luxS, lsrB, and lsrK mutants is likely due to impaired intracellular survival and systemic dissemination rather than defects in initial entry. In our intraperitoneal infection model, bacteria bypass the intestinal barrier, and successful colonization therefore depends largely on efficient survival and replication within host phagocytic cells.**

**We propose that the reduced colonization of these mutants is associated with diminished expression of SPI-2 encoded genes, which are essential for intracellular survival, replication within macrophages, and systemic spread. Loss of LuxS/AI-2 signaling or its uptake components may compromise proper regulation of SPI-2–dependent virulence pathways, thereby limiting bacterial persistence in host tissues. Consistent with this interpretation, the knockout strains exhibit reduced invasion and survival phenotypes, which together likely contribute to the observed attenuation in colonization following intraperitoneal infection.**

• In vivo, the luxS mutant is as attenuated as the lsrB and lsrK mutants in the intestine. As some members of the microbiota produce AI2, I would have expected that the luxS mutant would be less attenuated than the lsrB and lsrK mutants. Do you have any explanation?

**We thank the reviewer for this insightful comment. We also initially expected that the *luxS* mutant would be less attenuated than the *lsrB* and *lsrK* mutants in the intestine, given that AI-2 can be produced by members of the gut microbiota. However, our data show that the luxS mutant is similarly attenuated in vivo. One possible explanation is that, although AI-2 may be available in the intestinal environment, effective utilization of this signal depends on the functional expression and activity of the AI-2 uptake and signaling machinery. The *luxS* mutant may exhibit reduced expression or impaired regulation of AI-2 responsive pathways, limiting its ability to sense or respond to exogenous AI-2 produced by the microbiota. In contrast, bacteria naturally producing AI-2 are likely to possess active transport and signaling systems that efficiently internalize and process this molecule.**

**Thus, despite the presence of microbiota-derived AI-2, the *luxS* mutant may remain functionally impaired in AI-2 dependent regulation of virulence and fitness, resulting in an attenuation comparable to that observed for the *lsrB* and *lsrK* mutants.**

• Lines 548 and 549: incapable is too strong according to the phenotype you described. Please replace by “less able”

**We thank the reviewer for this suggestion and have replaced the term “incapable” with “less able” in lines 548–549 in the revised manuscript.**

• Lines 573-576: there is a misunderstanding here. SPI-2 effector secretion is triggered by the contact between the T3SS2 and the cytosol of eukaryotic cells (pH neutral) or if bacteria are exposed to pH7 after growth at ph5 (see ref 52), and not because the cytoplasm of bacteria is neutral.

**We thank the reviewer for pointing out this misunderstanding and for the helpful clarification. We agree that SPI-2 effector secretion is not triggered by the neutral pH of the bacterial cytoplasm, but rather by exposure to a neutral environment, such as contact of the T3SS2 with the host cell cytosol or a shift from acidic to neutral pH, as demonstrated in Ref. 52. We have revised the text to accurately reflect this mechanism. The revised version now clearly distinguishes between the requirement of acidic pH for SPI-2 apparatus assembly and the neutral pH–dependent triggering of effector secretion, and reframes the role of AI-2 signaling as indirectly supporting SPI-2 function by promoting bacterial fitness under acidic intracellular conditions.**

• Line 625: whenever

**Thank you so much for pointing out this mistake. We have corrected in revised manuscript.**

• AI-2 bioassays: it is not clear how the autoinducer assays were performed on infected macrophages. Was it the cell culture medium, a lysate of macrophages, a lysate of infected macrophages or something else that was used?

**We apologize for the lack of clarity in the original description of the AI-2 bioassays performed during macrophage infection. For the intracellular AI-2 assay, RAW 264.7 macrophages were infected, and cell lysates from infected macrophages were collected at different time points and subsequently used for the AI-2 bioassay. We have now revised the materials and methods section to explicitly describe this procedure and to avoid any ambiguity regarding the source of the samples used in these assays.**

• Lines 702-704: not understandable. Proposition: Then 20 μl of Salmonella cell-free culture supernatant was added to 180 μl of this diluted culture of the sensor strain?

**We thank the reviewer for pointing out the lack of clarity in this section. We have revised the text to explicitly state that 20 µL of Salmonella cell-free culture supernatant was added to 180 µL of the diluted V. harveyi BB170 sensor strain. This revision clarifies the experimental procedure and addresses the reviewer’s concern.**

• Line 709: control medium

**We thank the reviewer for noting this. We have corrected the term “media” to “medium”**

• Line 710 sterile medium

**We thank the reviewer for pointing this out. We have corrected “sterile media” to “sterile medium”.**

• Lines 756 and 759: please provide the reference of the antibodies and the dilutions used.

**The following antibody was used: LAMP1 monoclonal antibody (clone 1D4B; sc-19992, Santa Cruz Biotechnology, USA). The antibody was used at a dilution of 1:200 and incubated 2 hours at RT or overnight at 4℃.**

**The secondary antibody Alexa Fluor/DyLight 488–conjugated anti-rat IgG was used at a dilution of 1:200 and incubated for 1 h at room temperature.**

**We have now added this information to the revised manuscript.**

• The English of the paper would benefit from some editing.

**We thank the reviewer for this suggestion. The manuscript has been carefully revised, and the English language has been edited throughout to improve clarity, grammar, and readability.**
