# Supplementary material for: LuxS/AI-2 regulates phoP/phoQ by a non-canonical mechanism to enhance acid stress survival in Salmonella Typhimurium
Source: PLoS Pathog. 2026 May 28;22(5):e1014244. doi: 10.1371/journal.ppat.1014244 (PMC13218499; doi:10.1371/journal.ppat.1014244)
Supplement: Attachment — Submitted filename: Response to reviewers.docx [file ppat.1014244.s017.docx]

**Response to Reviewer’s comments for** **Manuscript Number: PPATHOGENS-D-25-02450R1**

PPATHOGENS-D-25-02450R1
LuxS/AI-2 regulates phoP/phoQ by a non-canonical mechanism to enhance acid stress survival in Salmonella Typhimurium
PLOS Pathogens

All changes made are highlighted in yellow in the revised manuscript, and the suggested text has been removed.

**Additional Editor Comments :**

Dear Dr. Chakravortty:

Thank you very much for submitting your manuscript "LuxS/AI-2 regulates phoP/phoQ by a non-canonical mechanism to enhance acid stress survival in Salmonella Typhimurium" (PPATHOGENS-D-25-02450R1) for review by PLOS Pathogens. Your revised manuscript was re-evaluated by the same peer reviewers. The reviewers appreciated the improvements and extensive experiments added to the manuscript but identified some aspects of the work that should still be improved. We therefore ask you to revise the manuscript according to the recommendations before we can consider your manuscript for acceptance. Your revisions should address the specific points made by each reviewer.

After reading the reviews and my own appreciation of the the original and the corrected manuscript, although no extra experimental work is required, I would like to note the two main things that still need to be addressed: (1) make sure that all modifications indicated in your last rebuttal are included in the manuscript, all figures are cited and all data is available and (2) that the proper statistical tests are used throughout the manuscript. In particular for this last point, please carefully consider the comments made by Reviewer 3 and the cited literature on this regards. After these items are addressed, I will be sending the manuscript for a final and second round of review. Please pay particular attention to the major and minor changes reviewer 3 suggested and give them due consideration. In addition, and to facilitate the next round of reviewing, please indicate in the rebuttal the lines that now contain the corresponding changes in the updated manuscript.

When you are ready to resubmit, please be prepared to provide the following:(1) A letter containing a detailed list of your responses to the review comments and a description of the changes you have made in the manuscript. (2) Two versions of the manuscript: one with either highlights or tracked changes denoting where the text has been changed; the other a clean version (uploaded as the manuscript file).

We hope to receive your revised manuscript within 60 days or less. If you anticipate any delay in its return, we ask that you let us know the expected resubmission date by replying to this email. If you have any questions or concerns while you make these revisions, please let us know.

Sincerely,

Camila Valenzuela, PhD
Guest Editor
PLOS Pathogens

Matthew C. Wolfgang, Ph.D.
Section Editor
PLOS Pathogens

**Thank you for your careful evaluation of our revised manuscript titled “LuxS/AI-2 regulates phoP/phoQ by a non-canonical mechanism to enhance acid stress survival in *Salmonella* Typhimurium” (PPATHOGENS-D-25-02450R1).**

**We sincerely appreciate the constructive feedback provided by you and the reviewers. We are grateful for the opportunity to further improve our manuscript.**

**Thank you again for your time and consideration. We look forward to submitting a thoroughly revised manuscript.**

**Journal Requirements:**

1) Please upload a copy of Figures 2, and 7 which you refer to in your text. Or, if the figures are no longer to be included as part of the submission please remove all reference to them within the text.

**Thank you for bringing this to our attention. We have now uploaded the revised versions of Figures 2 and 7 as separate files. Additionally, since modifications were made to other figures during revision, we have updated and re-uploaded all figures to ensure consistency throughout the manuscript.**

2) We have noticed that you have referred to supplementary tables and "Supplementary method" in the list of Supporting Information legends in your manuscript. However, there are no corresponding files uploaded to the submission. Please upload them as separate files with the item type 'Supporting Information'.

**Thank you for pointing this out, and we apologize for the confusion.**

**In our previous submission, the Supplementary Methods and all supplementary tables were included together in a single file. In the revised submission, we will upload the Supplementary Methods as a separate Supporting Information file and provide an individual separate document containing supplementary tables.**

**We will ensure that all files are properly labelled and correctly cited in the manuscript.**

3) Please ensure that the Data Availability Statements provided in the submission form and the manuscript are the same.

**Thank you for pointing this out. We have carefully checked and ensured that the Data Availability Statements in both the submission form and the manuscript are now identical and consistent.**

4) Please amend your detailed Financial Disclosure statement. This is published with the article. It must therefore be completed in full sentences and contain the exact wording you wish to be published.
1) State the initials, alongside each funding source, of each author to receive each grant. For example: "This work was supported by the National Institutes of Health (####### to AM; ###### to CJ) and the National Science Foundation (###### to AM)." 2) State what role the funders took in the study. If the funders had no role in your study, please state: "The funders had no role in study design, data collection and analysis, decision to publish, or preparation of the manuscript."

**Thank you so much for this. We have incorporated- The funders had no role in study design, data collection and analysis, decision to publish, or preparation of the manuscript.**

5) Please ensure that the funders and grant numbers match between the Financial Disclosure field and the Funding Information tab in your submission form. Note that the funders must be provided in the same order in both places as well.

**Thank you for highlighting this. We have carefully checked and ensured that the funders, grant numbers, and their order are consistent between the Financial Disclosure statement in the manuscript and the Funding Information section in the submission system.** 

Note: If the reviewer comments include a recommendation to cite specific previously published works, please review and evaluate these publications to determine whether they are relevant and should be cited. There is no requirement to cite these works unless the editor has indicated otherwise.


**Reviewers' Comments:**

Reviewer's Responses to Questions

**Part I - Summary**
Please use this section to discuss strengths/weaknesses of study, novelty/significance, general execution and scholarship.

Reviewer #1: The authors have addressed the comments of my previous review.

**We thank the reviewer for their positive evaluation of our revised manuscript and for acknowledging that the concerns raised in the previous review have been adequately addressed. We appreciate the reviewer’s time and constructive feedback, which helped improve the quality of our work.**

Reviewer #2: The authors addressed all my concerns, and I claim that the current version of the publication is almost ready for acceptance.

**We sincerely thank the reviewer for their positive evaluation of our revised manuscript. We are pleased that all concerns have been addressed. We appreciate the reviewer’s valuable feedback, which has significantly improved the quality of our work.**

Reviewer #3: I would like to thank the authors for their point-by-point answers to the Reviewer comments. Answers are very complete. I also really appreciate all the experiments that have been performed and added to the manuscript. My questions and concerns are listed below.

**We thank the reviewer for their careful evaluation of our manuscript and for their appreciation of our detailed point-by-point responses. We have carefully considered all the questions and concerns raised, and our detailed responses are provided below.**

**Part II – Major Issues: Key Experiments Required for Acceptance**
Please use this section to detail the key new experiments or modifications of existing experiments that should be absolutely required to validate study conclusions.

Reviewer #1: None

**We thank the reviewer for their evaluation of our manuscript, and we appreciate their time and consideration.**

Reviewer #2: The are no additional experiment needed.

**We thank the reviewer for their positive assessment and for noting that no additional experiments are required. We appreciate their time and thoughtful consideration of our manuscript.**

Reviewer #3: Major issues:
After careful reading of the manuscript, I am not sure that all the modifications mentioned by the authors in their answers have been included in the paper. I wonder if the version provided is the correct one (I read the yellow highlighted version) and I apologize if I missed several modifications.

**We sincerely apologize for confusion caused. We will carefully ensure that all the modifications mentioned in our responses are fully incorporated into the revised manuscript and clearly indicated. We appreciate the reviewer’s careful reading and will make every effort to avoid such inconsistencies in the updated version.**

A few examples concern the major issues of

(1) Reviewer 1 point 2 (I did not find changes announced by the authors in the discussion concerning this point),

**We sincerely apologize for this. As per the reviewer’s concern, we had moved the explanation of the experimental limitation from the Discussion to the Results section. However, we agree that this should also be addressed in the Discussion. We have now incorporated the relevant explanation in the Discussion section in the revised manuscript.**

**Added information- As noted earlier, a limitation of this study is that we cannot determine whether STM Δ*luxS* utilizes AI-2 produced by STM WT during coinfection in RAW 264.7 macrophages. In addition, our approach does not resolve whether both strains infect the same host cell, as the observations reflect a population-level phenotype. Future studies employing single-cell resolution approaches will be required to resolve these possibilities and further delineate AI-2 mediated interactions during infection. (Discussion-lines 606-612)**

(2) Reviewer 2 issues on figure 2G and line 234 (comparison not found in the results section) or on macrophages polarisation (did not find anything on this point in the discussion section)

**We thank the reviewer for pointing this out and apologize for the lack of clarity. With the reviewer 3 concern about this-We agree that our data do not support a definitive conclusion regarding upregulation in the absence of a neutral pH comparison. Accordingly, we have revised the statement to avoid overinterpretation. It now reads: “Our AI-2 bioassay in acidic medium shows that AI-2 production by Salmonella,” with emphasis on the observation that the AI-2 molecule remains stable under acidic pH conditions. (Line- 262)**

**In addition, we have incorporated the results (Fig. S9C-D) and discussion on macrophage polarisation in the Discussion section to address the reviewer’s concern. (Result section-lines 435-442) (Discussion-lines 589-598)**

(3) Reviewer 3 “Control of DNA absence in RNA samples” (where in the method section?)

**We sincerely apologize for this omission. The control for DNA contamination in RNA samples has now been incorporated in the methods section. (lines 836-839)**

(4) Reviewer 3 normalization relative to 2h (legends and methods).

**We thank the reviewer for this observation and apologize for the lack of clarity. The normalization relative to the 2 h time point has now been clearly specified in both the figure legends and the methods section. (lines- 846-850)**

Please make sure that all the modifications asked by the reviewers have been introduced.

**We have carefully ensured that all modifications suggested by the reviewers have now been incorporated into the revised manuscript.**

Statistical analyses: I am sorry but I do not really agree with the author’s answer to my concern about their statistical analyses. I agree with the authors that nonparametric tests are generally considered less powerful than parametric tests, but this does not justify the use of parametric tests if the assumptions for these latter tests are not met. The authors mention that they used parametric tests because their data follow a normal distribution (“symmetric”). How did the authors determine the normality of their data, given that statistical tests of normality are only useful for large samples (Van Belle G, Fisher LD, Heagerty PJ, Lumley T. Biostatistics: A Methodology for the Health Sciences. Hoboken, NJ: Wiley, 2004.)? Did they use normal probability plots? This could be a way to test normality for low sample size (Morgan CJ <https://doi.org/10.1152/ajplung.00238.2017>). Otherwise, permutation tests can be used, which appear to be as powerful as parametric tests and are suitable without the validity of the results relying on theoretical distributions.
I am not surprised that other papers in your research field used similar statistical tests as those you did. Most of us have used parametric tests incorrectly or without testing if the assumptions for these tests were met at some point in our careers. I believe that this should change for all of us. Please read the paper of (Olsen CH. 2014. Statistics in Infection and Immunity Revisited. Infect Immun 82:<https://doi.org/10.1128/iai.00811-13>), which provides a good overview of good and bad practices in statistics applied to our field of research.

**We thank the reviewer for their thoughtful and important comments regarding the statistical analyses. We fully agree that parametric tests should only be applied when their underlying assumptions are met, and that this is particularly critical when working with small sample sizes.**

**To address this concern, we have now explicitly evaluated the normality of our data using the Shapiro–Wilk test, which is considered appropriate and reliable for small sample sizes. The results of this analysis indicate that our datasets do not significantly deviate from normality (p > 0.05), supporting the use of parametric tests in our study.**

**In addition to formal testing, we also examined the distribution of the data using normal probability (Q–Q) plots, as recommended for small sample sizes (Morgan, 2017). These visual assessments further confirmed that the data are approximately normally distributed and symmetric.**

**We ensured that all data passed the Shapiro-Wilk test for normality. A few example data for Q-Q plots are shown here for reference.**

**Furthermore, we appreciate the suggestion regarding permutation tests. While permutation-based approaches are indeed powerful and assumption-free, the agreement between parametric and nonparametric results in our case provides confidence that our conclusions are not dependent on distributional assumptions.**

**Finally, we have carefully revised the manuscript to improve transparency in statistical reporting, in line with best practices highlighted in Olsen (2014).**

**Incorporated in method-** Normality of the data was assessed using the Shapiro–Wilk test. In addition, Q–Q plots were visually inspected to evaluate deviations from normality, particularly given the small sample size. Parametric tests were applied only when data did not significantly deviate from normality (p > 0.05) **(Lines-967-971)**

 

**Part III – Minor Issues: Editorial and Data Presentation Modifications**
Please use this section for editorial suggestions as well as relatively minor modifications of existing data that would enhance clarity.

Reviewer #1: None

Reviewer #2: The authors address all of my concerns. However, I have a question about the results and the statistical test used. There are many figures and experiments in which the authors compare 6 or 7 groups, with only three results per group, and they use ANOVA to do so. As far as I am concerned, to use ANOVA properly, you need at least N+1 biological replicates; for example, when comparing 7 groups, you should have at least 8 samples per group. Can the authors address my concern??

**We thank the reviewer for raising this important concern regarding the statistical analysis and sample size.**

**We agree that the relatively small number of biological replicates per group (n = 3) requires careful validation of the assumptions underlying parametric tests such as oneway ANOVA. In response, we have rigorously evaluated all datasets used in ANOVA for compliance with its core assumptions:**

**Normality: The distribution of each group was assessed using the *Shapiro–Wilk test*. In all cases, no significant deviation from normality was observed (p > 0.05), indicating that the data are approximately normally distributed.**

**Homogeneity of variance: We tested for equal variances across groups using Brown-Forsythe test. The results confirmed that the assumption of homoscedasticity was satisfied (p > 0.05).**

**Based on the validation of these assumptions, we proceeded with one-way ANOVA for comparisons involving multiple groups. Furthermore, when ANOVA indicated statistically significant differences, appropriate post hoc multiple comparison tests were performed to identify specific group differences.**

**We acknowledge that small sample sizes may limit statistical power; however, the use of ANOVA under validated assumptions is consistent with established practices in similar experimental studies (e.g., doi: 10.4103/aca.ACA_94_19).**

**We appreciate the reviewer’s insightful comment, which has helped us strengthen the rigor and clarity of our analysis.**

Reviewer #3: - If I am not mistaken, Supplementary tables are not provided into the pdf file provided to the Reviewers. It is thus not possible to verify that the new strains and oligos used to answer the reviewer comments have been added.

**We apologize for the inconvenience caused by the absence of the supplementary tables in the PDF provided for review.**

**In our submission, all supplementary materials, including the supplementary methods and tables, were combined into a single file. However, we understand that this format made it difficult to access and verify specific information. In accordance with the journal’s requirements, we have now revised the submission and uploaded the supplementary methods and supplementary table as separate files**. **The updated supplementary tables include all newly constructed strains and oligonucleotides used in this study. We hope that this revision resolves the issue and allows for clear verification of the added information.**

- Figure 7 is not cited in the text. It seems to correspond to a Graphical abstract but I do not see any graphical abstract in papers published in PLOS Pathogens. Is it new ? Is a legend necessary?

**We thank the reviewer for pointing out this oversight. We apologize that Figure 7 was not cited in the main text. We have now revised the manuscript to appropriately incorporate and cite Figure 7 at the relevant section.**

**Figure 7 is intended to represent a schematic summary of our findings (graphical overview). However, we acknowledge the reviewer’s concern regarding its classification as a graphical abstract, as this is not a standard requirement for *PLOS Pathogens*.**

**To address this, we have clarified the purpose of Figure 7 in the manuscript, presenting it as a schematic/model summarizing the study rather than as a formal graphical abstract. A detailed figure legend has now been included to improve clarity and interpretation.**

**We hope these revisions resolve the concern and improve the overall presentation of the manuscript.**

- Data availability statement: the authors have revised this section and state that all relevant data are within the manuscript and it Supporting Information files”. I am not sure this is the case. In most legends, the number of experiments and replicates is indicated (e.g., N=3 n=3) but the number of corresponding values is not provided in the figures (i.e. 9 values). Does this mean that figures were build using representative experiments, that some data were excluded or that all values are not presented and should therefore be added? Please look at all the legends and the corresponding figure or supplementary figure and provide an explanation for each of them or provide the missing data.

**We thank the reviewer for highlighting this important point and apologize for the lack of clarity in our initial presentation.**

**In our study, most experiments were performed with N = 3 or 4 independent biological replicates, with each replicate containing n ≥ 3 technical measurements. In several figures, we presented data from a single representative experiment (out of the three independent repeats), where each data point shown corresponds to individual technical measurements (e.g., n = 9 values in total). This was done to improve visual clarity, particularly in scatter plots.**

**To address the reviewer’s concern, we have now:**

1. **Explicitly clarified in all figure legends whether the data shown represent pooled results from all independent experiments or a single representative experiment.**
2. **Revised the Statistical Analysis section in the Methods to clearly define how N and n are used and how data are represented.**

**Importantly, no data were excluded from the analysis. All independent experiments yielded consistent results.**

**The full datasets underlying all figures (including all replicates) and supplementary information are available in the article or can be provided upon request from the corresponding author.**

**Revised Data Availability Statement**

**All relevant data are within the manuscript and its Supporting Information files. Additional datasets, including individual replicate values, are available from the corresponding author upon reasonable request.**

- Is S1B Fig cited somewhere? I did not find it in the text.

**We thank the reviewer for pointing out this oversight.**

**We apologize that Fig S1B was not cited in the main text. We have now carefully revised the manuscript and incorporated the appropriate citation to Figure S1B at the relevant section.**

- Fig 1A “Regulate multiple QS genes” instead of Regulate multiples genes-QS genes” and “phosphorylated AI-2 binds LsrR and removes it from lsr promoter” instead of “phosphorylated AI-2 binds LsrR and remove it from lsr promoter”

**We thank the reviewer for carefully noting these textual errors in Figure 1A.We have corrected the labels as suggested:**

**“Regulate multiples genes-QS genes” has been revised to “Regulate multiple QS genes”.**

**“phosphorylated AI-2 binds LsrR and remove it from lsr promoter” has been revised to “phosphorylated AI-2 binds LsrR and removes it from the lsr promoter”.**

**These corrections have been incorporated into the revised figure.We appreciate the reviewer’s attention to detail, which has helped improve the clarity and accuracy of the figure.**

- Concerning my comment on the fold expression to 1 at 2h post-infection. Thank you for the explanations that are clear. In this case, I would suggest to change the title of the y axis of the graphs of the corresponding figures. For example, “relative expression of “gene X”/2h pi”

**We thank the reviewer for this helpful suggestion and for acknowledging our clarification regarding normalization at 2 h post-infection. As recommended, we have revised the y-axis labels of the relevant graphs to clearly indicate the normalization reference point. The labels now read, for example: “Relative expression of gene X / 2 h post-infection (pi)” or Relative gene expression to 3h. This modification improves the clarity and ensures that the normalization strategy is explicitly communicated to the reader.**

**We appreciate the reviewer’s suggestion, which has enhanced the presentation of our data.**

- I would like to thank the authors for including the paper of Thijs in the discussion part.

**We thank the reviewer for their positive feedback and are pleased that the inclusion of the study by Thijs has strengthened the discussion.**

According to EMSA results, it seems to me that the authors observe LsrR binding to both the single and the double-stranded DNA of the phoPQ promoter. Am I wrong? If not, I am not sure that the single strand interaction observed by the authors could explain the difference obtained in the two studies.

**We thank the reviewer for this insightful observation and agree with their interpretation. Our EMSA results indicate that LsrR binds both single-stranded and double-stranded DNA of the *phoP/Q* promoter *in vitro*. However, our in silico analysis consistently predicts a stronger and more specific interaction with the single-stranded *phoP* promoter (involving nucleotide sequence of the single strand), suggesting a possible preference under certain conditions. We acknowledge that the *in vitro* EMSA results do not fully distinguish this preference and instead show binding to both forms. Based on these observations, we propose that the interaction of LsrR with the single-stranded *phoP* promoter may be sufficiently strong to facilitate detectable binding to both forms.Importantly, we note that *in vitro* binding assays (such as EMSA) may not fully recapitulate the physiological context, where DNA structure, supercoiling, and protein cofactors can influence binding specificity. Therefore, we believe that the apparent discrepancy between our findings and previous studies may arise from differences in experimental conditions and DNA conformational states.**

**To address this concern and avoid overinterpretation, we have:**

1. **Revised our conclusions to state that LsrR is “*predicted”* to preferentially interact with the single-stranded *phoP* promoter based on in silico analysis, rather than making a definitive claim. (Lines- 390-393)**
2. **Clearly reported the EMSA findings in our results, showing binding to both single- and double-stranded DNA.**
3. **Expanded the Discussion to explicitly acknowledge this discrepancy and propose targeted future experiments (e.g., *in vivo* binding assays or structure-specific binding studies) to further solve the enigma of LsrR binding. (Lines 624-639)**

**We hope this clarification addresses the reviewer’s concern and improves the transparency of our interpretation.**

- Line 117: please add that DPD is secreted by Salmonella.

**We thank the reviewer for this helpful suggestion. We have revised the sentence at line 116 to explicitly indicate that DPD is secreted by Salmonella. This clarification has been incorporated into the revised manuscript to improve accuracy and completeness**.

- Lines 171-174 : I agree that in S1G figure, the data suggest that bacteria reside in a vacuole as LAMP1 colocalize with the strains. I am not convinced for the S1H figure, especially for the lsrR mutant. Numerous bacteria do not colocalize with LAMP1. This picture looks like cytosolic hyper-replicative bacteria that have already been described in RAW 264.7 cells by Roder and Hensel 2020(DOI: 10.1111/cmi.13155). As only a few cells are shown, we cannot exclude that cytosolic hyper-replication could also be observed for the other strains as this phenomenon is rare (about 10% of the infected cells).

**We thank the reviewer for this insightful comment and for highlighting the study by Roder and Hensel (2020). We agree that in Figure S1G, LAMP1 colocalization supports a predominantly vacuolar localization of bacteria. Regarding Figure S1H, we acknowledge that a subset of bacteria—particularly in the lsrR mutant do not colocalize with LAMP1. As suggested by the reviewer, these bacteria could represent cytosolic populations. However, we would like to emphasize that cytosolic hyper-replication of Salmonella is highly cell-type dependent**.

**The study by Roder and Hensel 2020** **was performed in epithelial (HeLa) cells, where cytosolic hyper-replication is well documented. In contrast, our experiments were conducted in RAW 264.7 macrophages, which present a markedly different intracellular environment. Several studies have shown that, unlike epithelial cells, the macrophage cytosol is generally restrictive for Salmonella survival and replication, with host defense mechanisms actively limiting cytosolic bacteria (Castanheira and García-del Portillo, 2017; Egan et al., 2024). Furthermore, Salmonella is well established to adopt a predominantly vacuolar lifestyle within macrophages, which supports its intracellular survival (Richardson, 2015).**

**We sincerely thank the reviewer for this insightful comment. We acknowledge the significance of this point and will consider it as an important direction for future investigation**.

- Line 177 please add compared to 2h pi

**We have added this information to the revised manuscript.**

- Figure 2 legend: for H, I and J, please provide N and n

**We have revised the Figure 2 legend to include the number of independent experiments (N) and replicates (n) for panels H, I, and J.**

- In my opinion, Figure 2G does not allow to conclude that acidification is a physiological signal that upregulates AI-2 production by Salmonella (line 265). A comparison with data obtained after growth at neutral pH in the same medium is needed. Please clarify the use of the term “up-regulation” in line 265.

**We thank the reviewer for this important comment. We agree that our data do not support a definitive conclusion regarding upregulation in the absence of a neutral pH comparison. Accordingly, we have revised the statement to avoid overinterpretation. It now reads: “Our AI-2 bioassay in acidic medium shows that AI-2 production by Salmonella,” with emphasis on the observation that the AI-2 molecule remains stable under acidic pH conditions.**

Moreover, the experiments performed with Vibrio allow the amount of AI-2 in the supernatant of Salmonella culture to be evaluated. I agree that the amount of AI2 present in the supernatant is maximal during the mid- and late-log phases but the amount of AI2 produced by Salmonella is probably not maximal at these two phases, as the number of bacteria secreting AI2 in the supernatant is higher during these growth phases than in the lag-phase and in the early logarithmic phase. Therefore, I am not sure that the statement “Moreover, we observed that STM stably produces AI2 even during its growth in F-medium, which is maximal during the mid to late-log phase” is true. In my opinion, it is important to distinguish between the amount of AI2 present in the supernatant and the amount of AI2 produced by Salmonella. Expressing the results in RLU/OD, as requested by another reviewer, answers the question of how much AI2 is produced by Salmonella and is a complementary information to the amount of AI2 present in the culture supernatant.

**We thank the reviewer for this insightful comment and agree that it is important to distinguish between the amount of AI-2 present in the culture supernatant and the amount produced by Salmonella per cell. The Vibrio-based bioassay primarily reflects the accumulated AI-2 in the supernatant, which is influenced by both production and bacterial density across growth phases.**

**As noted previously, normalization to RLU/OD₆₀₀ can, in principle, provide a measure of AI-2 production per cell. However, in our experimental setup, this normalization introduced disproportionate variability and did not reliably capture AI-2 dynamics, likely due to the continuous accumulation and turnover of AI-2 in the medium. Notably, we also observed a reduction in AI-2 signal after 6 h, suggesting that AI-2 levels are not directly proportional to cell density and may reflect additional factors such as stability or, more importantly, uptake.**

**Accordingly, we have revised the text to avoid overinterpretation and to clearly state that our measurements reflect AI-2 levels in the supernatant rather than direct per-cell production rates. (Line 272)**

- Figure S4 and Lines 296 to 304: The different parts of this figure are not correctly referenced in the text. Figures 4A to 4C do not compare the WT and the mutants, Figure 4D is not related to F-medium, Figure 4G is not a comparison of phoP and phoQ expression in the WT and mutants upon infection of macrophages. Please correctly reassign the figures to the corresponding text.

**We thank the reviewer for carefully pointing this out. The figure panels were incorrectly referenced in the original version. We have now corrected and reassigned the figure panels to accurately correspond with the respective descriptions in the text.**

- Line 324: measured instead of validated

**We thank the reviewer for the suggestion; “validated” has been replaced with “measured” in the revised manuscript.**

- Line 325: please add “and STM WT “

**We thank the reviewer for the suggestion; “and STM WT” has now been added in the revised manuscript.**

- Line 326: please add “and observed diminished…”

**We thank the reviewer for the suggestion; “and observed diminished” has been incorporated into the revised manuscript.**

- Fig 3 B legend: please precise the medium used

**We thank the reviewer for the suggestion; the medium used in Fig. 3B has now been clearly specified in the revised legend.**

- Line 388: According to quantifications provided in FigS6, Y25A alone and R43A alone do not seem to reduce the binding of LsrR compared to WT LsrR in most cases. What data do the authors base the assertion that Y25A and R43A showed reduced binding on?

**We sincerely apologize for this oversight. Our initial statement was based on in silico predictions; however, we acknowledge that we do not have direct experimental evidence to support reduced binding for the Y25A and R43A mutants. Accordingly, we have removed this overinterpreted statement and revised the manuscript to accurately reflect the available data.**

- Lines 392-394 and Lines 395-397: duplicate entry?

**We thank the reviewer for pointing this out; the duplicated text has been removed and the paragraph has been revised accordingly.**

- Figure 4 legend: C and D please specify if EMSA were performed with double or single strand DNA.

**We thank the reviewer for the suggestion; double-stranded DNA used in EMSA for Fig. 4C and 4D has now been specified in the revised legend.**

- Figure S10 and S11: what does gm (y axis) means? And what is shown the Mice weight reduction (S10 legend) or mice weight (S11 legend and S10E-F and S11F figures)?

**We thank the reviewer for pointing this out; “gm” represents grams, indicating mouse body weight, and this has now been clarified in the revised figures/legends.**

**We thank the reviewer for highlighting this ambiguity; the data represent mouse body weight (in grams), not weight reduction, and the figure legends have been revised accordingly for clarity.**

PLOS authors have the option to publish the peer review history of their article ([what does this mean?](https://track.editorialmanager.com/CL0/https:%2F%2Fjournals.plos.org%2Fplospathogens%2Fs%2Feditorial-and-peer-review-process%23loc-peer-review-history/1/010f019cf6ab6ccf-f2f2b010-2c80-43b9-8521-fcab0fef0457-000000/QPzaeveWsFNTvnqAUnPHqaifOUgkHlgiSc5rwSTAhsA=254)). If published, this will include your full peer review and any attached files.

If you choose “no”, your identity will remain anonymous but your review may still be made public.

**Do you want your identity to be public for this peer review?** For information about this choice, including consent withdrawal, please see our [Privacy Policy](https://track.editorialmanager.com/CL0/https:%2F%2Fwww.plos.org%2Fprivacy-policy/1/010f019cf6ab6ccf-f2f2b010-2c80-43b9-8521-fcab0fef0457-000000/bdJyTUsI-EB_IPzikQ-yUxGL3pC2jlZ_kriGRbGQEVo=254).

Reviewer #1: No

Reviewer #2: No

Reviewer #3: No

[NOTE: If reviewer comments were submitted as an attachment file, they will be attached to this email and accessible via the submission site. Please log into your account, locate the manuscript record, and check for the action link "View Attachments". If this link does not appear, there are no attachment files.]

**Figure resubmission:**

While revising your submission, we strongly recommend that you use PLOS’s NAAS tool (https://ngplosjournals.pagemajik.ai/artanalysis) to test your figure files. NAAS can convert your figure files to the TIFF file type and meet basic requirements (such as print size, resolution), or provide you with a report on issues that do not meet our requirements and that NAAS cannot fix.

After uploading your figures to PLOS’s NAAS tool - <https://ngplosjournals.pagemajik.ai/artanalysis>, NAAS will process the files provided and display the results in the "Uploaded Files" section of the page as the processing is complete. If the uploaded figures meet our requirements (or NAAS is able to fix the files to meet our requirements), the figure will be marked as "fixed" above. If NAAS is unable to fix the files, a red "failed" label will appear above. When NAAS has confirmed that the figure files meet our requirements, please download the file via the download option, and include these NAAS processed figure files when submitting your revised manuscript.

**Reproducibility:**

To enhance the reproducibility of your results, we recommend that authors of applicable studies deposit laboratory protocols in protocols.io, where a protocol can be assigned its own identifier (DOI) such that it can be cited independently in the future. Additionally, PLOS ONE offers an option to publish peer-reviewed clinical study protocols. Read more information on sharing protocols at https://plos.org/protocols?utm_medium=editorial-email&utm_source=authorletters&utm_campaign=protocols
