# Supplementary material for: LuxS/AI-2 regulates phoP/phoQ by a non-canonical mechanism to enhance acid stress survival in Salmonella Typhimurium
Source: PLoS Pathog. 2026 May 28;22(5):e1014244. doi: 10.1371/journal.ppat.1014244 (PMC13218499; doi:10.1371/journal.ppat.1014244)
Supplement: Attachment — Submitted filename: 2) Response to Reviewers.docx [file ppat.1014244.s018.docx]

**Response to Reviewer’s comments for** **Manuscript Number: PPATHOGENS-D-25-02450R2**

PPATHOGENS-D-25-02450R2
LuxS/AI-2 regulates phoP/phoQ by a non-canonical mechanism to enhance acid stress survival in Salmonella Typhimurium
PLOS Pathogens

Dear Dr. Chakravortty,

Thank you for submitting your manuscript to PLOS Pathogens. After careful consideration, we feel that it has merit but does not fully meet PLOS Pathogens's publication criteria as it currently stands. Therefore, we invite you to submit a revised version of the manuscript that addresses the points raised during the review process.

Please submit your revised manuscript by Jun 27 2026 11:59PM. If you will need more time than this to complete your revisions, please reply to this message or contact the journal office at plospathogens@plos.org. When you're ready to submit your revision, log on to <https://www.editorialmanager.com/ppathogens/> and select the 'Submissions Needing Revision' folder to locate your manuscript file.

Please include the following items when submitting your revised manuscript:

* A letter that responds to each point raised by the editor and reviewer(s). You should upload this letter as a separate file labeled 'Response to Reviewers'. This file does not need to include responses to any formatting updates and technical items listed in the 'Journal Requirements' section below.
* A marked-up copy of your manuscript that highlights changes made to the original version. You should upload this as a separate file labeled 'Revised Manuscript with Track Changes'.
* An unmarked version of your revised paper without tracked changes. You should upload this as a separate file labeled 'Manuscript'.

If you would like to make changes to your financial disclosure, competing interests statement, or data availability statement, please make these updates within the submission form at the time of resubmission. Guidelines for resubmitting your figure files are available below the reviewer comments at the end of this letter.

We look forward to receiving your revised manuscript.

Kind regards,

Camila Valenzuela
Guest Editor
PLOS Pathogens

Matthew Wolfgang
Section Editor
PLOS Pathogens

Sumita Bhaduri-McIntosh
Editor-in-Chief
PLOS Pathogens
orcid.org/0000-0003-2946-9497

Michael Malim
Editor-in-Chief
PLOS Pathogens
orcid.org/0000-0002-7699-2064

**Dear Editor,**

**Thank you for your email and for the careful evaluation of our manuscript. We sincerely appreciate the time and effort you and the reviewers invested in assessing our work.**

**We are grateful for the opportunity to revise our manuscript for further consideration in PLOS Pathogens. We appreciate the constructive comments and suggestions provided during the review process, which we believe will help us to improve the quality and clarity of the manuscript.**

**We carefully addressed all the reviewers' points and submitted a thoroughly revised version of the manuscript within the stipulated timeline.**

**Thank you again for your consideration.**

All changes made are highlighted in yellow in the revised manuscript, and the suggested text has been removed.

Additional Editor Comments:

Dear Dr. Chakravortty:

Thank you very much for your re-revised version of the manuscript "LuxS/AI-2 regulates phoP/phoQ by a non-canonical mechanism to enhance acid stress survival in Salmonella Typhimurium" (PPATHOGENS-D-25-02450R2). Your revised manuscript was re-evaluated by two of the original peer reviewers, who agreed that the modifications made address almost all aspects discussed during the previous rounds of revision. After carefully reading the minor comments from Reviewer 3, I have come to the conclusion that these data visualization changes should be incorporated before we can formally accept this manuscript. Therefore, I am recommending a Minor Revision, after which I will personally evaluate how the changes were implemented, without sending it to the original reviewers, and provisionally accept the new version.

**We sincerely appreciate the time and effort taken by you and the reviewers throughout the review process. We are grateful for the positive assessment that the revisions have addressed almost all concerns raised in the earlier rounds.**

**We also thank Reviewer 3 for the additional minor suggestions regarding data visualization. We appreciate the opportunity to further improve the manuscript and have carefully incorporated all the requested changes in the revised version.**

**Thank you once again for your consideration and support.**

Journal Requirements:

1) We have noticed that you have uploaded Supporting Information files, but you have not included a complete list of legends. Please add a full list of legends for your Supporting Information file (Supporting Information.docx) after the references list.

**We sincerely apologize for the oversight regarding the incomplete list of legends in the Supporting Information file. We have incorporated legends in the supporting information files after the reference list. (Line- 148, in supporting information file)**

**We have now carefully revised the Supporting Information.docx file and incorporated a complete list of legends**

2) Please ensure that the funders and grant numbers match between the Financial Disclosure field and the Funding Information tab in your submission form. Note that the funders must be provided in the same order in both places as well.

**We carefully checked the Financial Disclosure field and the Funding Information tab in the submission form, and we ensured that the funders and corresponding grant numbers are presented consistently in the same order in both sections.**

**We appreciate your guidance in helping us maintain accuracy in the submission.**

 Note: If the reviewer comments include a recommendation to cite specific previously published works, please review and evaluate these publications to determine whether they are relevant and should be cited. There is no requirement to cite these works unless the editor has indicated otherwise.


Reviewers' Comments:

Reviewer's Responses to Questions

Part I - Summary
Please use this section to discuss strengths/weaknesses of study, novelty/significance, general execution and scholarship.

Reviewer #2: In my opinion the paper is ready for acceptance.

**We sincerely thank Reviewer #2 for the positive evaluation of our manuscript and for considering the paper ready for acceptance. We greatly appreciate the reviewer’s time, support, and encouraging assessment of our work.**

Reviewer #3: I would like to thank the authors for taking into account all the Reviewer comments and for their detailed answers in this R2 version. I have now only a few minor concerns

**We sincerely thank Reviewer #3 for the positive and encouraging comments. We greatly appreciate the reviewer’s careful reassessment of our revised manuscript and are grateful that our responses and revisions have satisfactorily addressed the major concerns raised in the previous rounds.**

**We also thank the reviewer for the remaining minor suggestions, which further improved the quality and presentation of the manuscript. All points have been carefully addressed in the revised version.**

Part II – Major Issues: Key Experiments Required for Acceptance
Please use this section to detail the key new experiments or modifications of existing experiments that should be absolutely required to validate study conclusions.

Generally, there should be no more than 3 such required experiments or major modifications for a "Major Revision" recommendation. If more than 3 experiments are necessary to validate the study conclusions, then you are encouraged to recommend "Reject".

Reviewer #2: none.

**We appreciate the reviewer’s assessment that no major issues or additional key experiments are required for acceptance. We are grateful for the reviewer’s positive support and valuable time throughout the review process.**

Reviewer #3: None

**We sincerely thank Reviewer #3 for the careful evaluation of our manuscript. We greatly appreciate the reviewer’s assessment that no major issues or additional experiments are required. We are grateful for the reviewer’s constructive comments and continued support throughout the review process.**

 Part III – Minor Issues: Editorial and Data Presentation Modifications
Please use this section for editorial suggestions as well as relatively minor modifications of existing data that would enhance clarity.

Reviewer #2: none

**We sincerely thank Reviewer #2. We truly appreciate the reviewer’s view that no additional minor revisions or editorial changes are necessary. We are grateful for the reviewer’s continued encouragement and positive assessment of our work.**

Reviewer #3: • The availability of the data is not still very clear to me, even though many details have been added. I would like to give a few examples, but this issue recurs throughout the article:

**We sincerely thank Reviewer #3 for this important observation regarding data availability and clarity. We apologize that the presentation of the data was still not sufficiently clear in certain sections despite our previous revisions. We appreciate the reviewer for highlighting this recurring concern. In response, we have carefully re-examined the manuscript throughout and further revised the relevant sections to improve clarity, consistency, and transparency in data presentation. The reviewer's specific examples have been addressed individually below.**

- In Figure S5 ABC, there are only three data points per histogram, whereas in the legend, the authors wrote, “All data are represented as… (N=3, n=3).”

**We thank the reviewer for this. We would like to clarify that the data presented in Figure S5A–C represent three independent experiments (N = 3). In each experiment, measurements were performed in triplicate (n = 3), and the values were averaged within each experiment. The histogram therefore displays three data points, each corresponding to the mean value from a separate independent experiment. That’s why the figure legend is-** All data are represented as mean ± SD from independent experiments (N=3, n=3). **(Lines- (1096-1097)**

- In Figure 1E, numerous points are shown on each histogram, but in the legend, the authors wrote “the data come from an experiment representative,” which implies that only 3 points should be present,

**We are very sorry for creating the confusion. As in the previous revised manuscript, we have mentioned that some data are representative. In the expression study of the genes in Figure 1D and 1E, we represented 9 data value point as we followed the following analysis-**

**For data point-**

**For example, only-**

**1) RAW Ct value-**

**Ct values were obtained for 16S rRNA (internal control) and gene A (Gene of interest).**

**Measurements were taken at 2 h, 6 h, and 16 h, with three technical replicates at each time point.**

**2) Averaging Ct values –**

**Calculated the mean Ct for 16S rRNA at each time point**

**3) ΔCt calculation (normalization to 16S rRNA)-**

**For each technical replicate and for the same time point, we normalized Gene A to 16S rRNA:** $\boldsymbol{\Delta}\boldsymbol{Ct}\mathbf{=}\boldsymbol{C}\boldsymbol{t}_{\text{Gene A}}\mathbf{-}\boldsymbol{C}\boldsymbol{t}_{\text{16}\text{S rRNA}}$

**4) ΔΔCt calculation (normalization to reference condition)-**

**Next, we normalized ΔCt values to a reference condition (e.g. WT an early time point such as 2 h):**

$$\boldsymbol{\Delta\Delta}\boldsymbol{Ct}\boldsymbol{=\Delta}\boldsymbol{C}\boldsymbol{t}_{\text{sample}}\mathbf{-}\boldsymbol{\Delta}\boldsymbol{C}\boldsymbol{t}_{\text{reference}}$$

**Since we normalized each** $\boldsymbol{\Delta}\boldsymbol{Ct}$ **value of each time point to each** $\boldsymbol{\Delta}\boldsymbol{Ct}$ **value of 2h ( 3x3), we are getting 9 data point for each data time point.**

**5)Fold-change calculation-**

**Relative expression was calculated using:**

$$\text{Fold}\text{ change}\mathbf{=}\mathbf{2}^{\mathbf{-}\boldsymbol{\Delta\Delta}\boldsymbol{Ct}}$$

**We revised the manuscript and clearly stated that the data are representative of N=3, n=3. Also, most of the data is represented as mean ± SD**

- In Figures 1F and 1H, only 3 points are present for each histogram, but upon reading the legend, it seems to me that all the data should have been provided.

**We thank the reviewer for highlighting this point and apologise for the confusion regarding the number of plotted data points in Figures 1F and 1G.**

**For Figure 1F, phagocytosis was calculated from a single pre-inoculum of bacterial culture, with 3 technical replicates at 2h. So, in Figure 1H, there are three points.**

**The apparent discrepancy between the stated number of technical replicates (n) and the number of plotted data points arises from the normalisation strategy used for fold change calculations (Figure 1G). Specifically, measurements obtained at 16 h were normalised against all corresponding 2 h measurements, rather than to a single paired value. This approach was intentionally adopted to avoid bias associated with one-to-one normalisation, as the 2 h and 16 h samples were collected from independent wells rather than from the same culture over time. As a consequence, each 16 h measurement generates multiple fold-change values (9 data points) when normalised against multiple independent 2 h measurements. This results in more plotted data points without increasing the number of biological replicates.**

**Furthermore, the figures present representative data from one of three independent biological experiments, which may contribute to the perceived discrepancy. Importantly, consistent trends were observed across all biological replicates.**

**For Figure 1H, the displayed data correspond to a representative experiment in which four technical replicates (n = 4) were performed. We have now explicitly clarified this in the revised figure legend and clearly defined N (number of independent biological replicates) and n (number of technical replicates) for all experiments. We revised the legend to clearly mention the representative data with N and n. All changes lines- 185-197.**

- In Figure 2, I do not see any histogram with more than 4 data points, but in the corresponding legend, the authors did not always write “The data are from a representative experiment of 3 independent experiments, N=3, n=4.” Instead, at the end of the legend for Figure 2, they wrote, “All data are presented as mean ± standard deviation from three independent experiments.”

**We sincerely apologise for this oversight and thank the reviewer for bringing it to our attention. In the last revised manuscript, we have indicated which data are representative. In addition, we have specified that all data are presented as mean ± SD from three independent biological experiments for merged data.**

**We acknowledge the reviewer's point that the legend in Figure 2 is confusing. In response, we revised the Figure Legend for clarification. To address this, we have revised the figure legend to improve clarity and ensure consistency in data representation. (Lines- 238-250)**

• Regarding Figure S1H and the colocalization (or not of Salmonella) with LAMP-1, I do not share the authors’ view. Rôder and Hensel 2020 did not work exclusively with epithelial HeLa cells in their paper as stated by the authors. They also tested RAW 264.7 macrophages and wrote: “Time-lapse microscopy of RAW264.7 cells infected with STM harbouring the dual colour reporter revealed intracellular proliferation of STM WT (Movie 3) and occasionally the appearance of host cells with sfGFP-expressing cytosolic bacteria … (Movie 4)”. These cytosolic Salmonella are observable using confocal microscopy thus under the conditions used by Singh et al.. We confirmed the results of Röder and Hensel on RAW 264.7 cells in our lab using both the reporter plasmid used by Röder and Hensel and LAMP-1 and Salmonella colocalization experiments, as Singh et al did.
The S1H picture shows a lack of LAMP-1 colocalization with numerous Salmonella for the lsrR mutant and a typical picture of cytosolic hyper-replication we observe when performing this type of experiment on RAW 264.7 experiments. I therefore agree with the authors that “Salmonella adopt a predominantly vacuolar life style within macrophages”, but Salmonella also adopts a cytosolic lifestyle in macrophages. The lack of LAMP-1 colocalization with Salmonella in figure S1H for the lsrR mutant therefore does not allow to conclude that Salmonella is vacuolar for this mutant (Line 173-174). And, as I mentioned in my review of version R1, “As only a few cells are shown in FigS1H for all the strains tested, we cannot exclude that cytosolic hyper-replication could also be observed for other strains than the lsrR mutant as cytosolic Salmonella is rare phenomenon (about 10% of the infected cells in our hands)”. In conclusion, in my opinion, the authors cannot claim that “all the strains reside in SCV” (Lines 173-174) based on the confocal microscopy images presented in figure S1H.

**We sincerely thank Reviewer #3 for this careful and insightful comment. We apologize for our earlier overinterpretation of the confocal microscopy data and appreciate the reviewer for highlighting the important distinction between predominant vacuolar localization and the possibility of cytosolic *Salmonella* in macrophages.**

**We agree that the images presented in Figure S1H do not allow a definitive conclusion that all strains exclusively reside within SCVs. We also acknowledge that cytosolic *Salmonella* may occur in macrophages, although as a relatively infrequent event. In addition, based on our microscopy observations, the knockout strains appeared to proliferate less efficiently in comparison with the STM WT under the tested conditions. The corresponding statements in the manuscript have been modified accordingly to better reflect the limitations of the data and the reviewer’s valuable suggestion. (Lines-167-169)**

• Line 58 : please do not italicize Typhimurium

**We thank the reviewer for noting this formatting error. The italicization of Typhimurium at Line 56 has been corrected in the revised manuscript.**

• Line 136: please remove one of the two “the and add the notion that LsrR binds to both single and double strand phoP/phoQ promoter DNA

**The duplicated word “the” at Line 133 has been removed. In addition, the sentence has been revised to clarify that LsrR binds to both single and double-stranded phoP/phoQ promoter DNA. We thank the reviewer for this careful observation.**

• Line 269 I suppose that the authors wanted to write “Our AI-2 bioassay in acidic medium shows AI-2 production by Salmonella

**We thank the reviewer for identifying this wording issue. The sentence has been corrected and now reads: Our AI-2 bioassay in acidic medium shows AI-2 production by *Salmonella*. (Lines 262)**

• Line 275-279: please rewrite which is long and not clear.

**We thank the reviewer for this helpful comment. We agree that the original sentence was lengthy and unclear. The text in lines has been rewritten for improved clarity and readability in the revised manuscript. (lines- 269-276)**

• Line 301303: Thank you for having added FigS4A-D. Please please add at least a short interpretation of the results shown in these figures

**We thank the reviewer for this valuable suggestion. We have now included a brief interpretation of the results presented in Fig. S4A–D in the revised manuscript to improve clarity. (Lines-297-300)**

• FigS2C: I would suggest to add DPD after the μM concentration on the x axis

**We thank the reviewer for this helpful suggestion. We have revised Fig. S2C to include “DPD” after the μM concentration on the x-axis for improved clarity.**

• FigS2I x axis: I would suggest to write BF-8 instead of BF

**We thank the reviewer for this suggestion. The x-axis label in Fig. S2H and S2I has been corrected from “BF” to “BF-8” in the revised figure.**

• Table S1: please provide bibliographic reference instead of “Laboratory stock” as sources in Table S1 so that scientists reading your paper can access the history of the plasmids and strains you used

**We thank the reviewer for this important suggestion. We agree that providing proper bibliographic references enhances the accessibility and reproducibility of the work. Accordingly, we have revised Table S1 to “Laboratory stock” with appropriate references for all plasmids and strains wherever available.**

• Fig S10 and 11: if the weight is expressed in grams, then I would suggest to use the international nomenclature i.e. g instead of gm

**We thank the reviewer for this helpful suggestion. The unit has been corrected from “gm” to the standard SI notation “g” in Figure 6 and S10 and S11 in the revised manuscript.**
